# Current and future outlook of loaded components in hydrogel composites for the treatment of chronic diabetic ulcers

**DOI:** 10.3389/fbioe.2023.1077490

**Published:** 2023-02-13

**Authors:** Jiaming Cui, Siqi Zhang, Songmiao Cheng, Hai Shen

**Affiliations:** ^1^ Sichuan Provincial Orthopaedic Hospital, Chengdu, Sichuan, China; ^2^ Guangzhou Medical University, Guangzhou, Guangdong, China

**Keywords:** hydrogel composites, chronic diabetic ulcers, biological properties, biomedical applications, wound healing

## Abstract

Due to recalcitrant microangiopathy and chronic infection, traditional treatments do not easily produce satisfactory results for chronic diabetic ulcers. In recent years, due to the advantages of high biocompatibility and modifiability, an increasing number of hydrogel materials have been applied to the treatment of chronic wounds in diabetic patients. Research on composite hydrogels has received increasing attention since loading different components can greatly increase the ability of composite hydrogels to treat chronic diabetic wounds. This review summarizes and details a variety of newly loaded components currently used in hydrogel composites for the treatment of chronic diabetic ulcers, such as polymer/polysaccharides/organic chemicals, stem cells/exosomes/progenitor cells, chelating agents/metal ions, plant extracts, proteins (cytokines/peptides/enzymes) and nucleoside products, and medicines/drugs, to help researchers understand the characteristics of these components in the treatment of diabetic chronic wounds. This review also discusses a number of components that have not yet been applied but have the potential to be loaded into hydrogels, all of which play roles in the biomedical field and may become important loading components in the future. This review provides a “loading component shelf” for researchers of composite hydrogels and a theoretical basis for the future construction of “all-in-one” hydrogels.

## 1 Introduction

The prevalence of diabetes, a chronic disease that poses a serious health threat, is rapidly increasing (Khursheed et al., 2019; Gao et al., 2021b). According to the International Diabetes Federation, 460 million adults worldwide now have diabetes, and this number is expected to rise to more than 700 million by 2045 (Li et al., 2022c). With the steep rise in the global prevalence of diabetes, the prevalence of its complications, such as chronic wounds and ulcers, has also increased tremendously (Holl et al., 2021). The most common and serious of these complications, diabetic foot ulcers (with a prevalence up to 25%) (Zheng et al., 2019), are the most important cause of amputation and reamputation in diabetic patients (Mishra et al., 2017), with a mortality rate of more than 50% within 5 years after amputation (Kimball et al., 2017). Therefore, the risk of amputation and the poor prognosis of refractory diabetic wounds not only impose a substantial burden on patients and their families but also represent a major challenge for orthopedic clinicians and even global health systems (Han and Ceilley, 2017; Theocharidis et al., 2022).

Currently, the treatment strategies for diabetic chronic wounds and ulcers include debridement, infection control, and local physiotherapy (Chung et al., 1998; Zhou et al., 2022). However, due to the effects of recalcitrant microangiopathy and chronic infections, traditional treatments do not easily produce satisfactory healing effects for chronic wounds or ulcers in diabetic patients (Wei et al., 2021); thus, this problem has troubled clinicians for a long time. Therefore, considering the characteristics of diabetic wounds, the development of more rapid and efficient therapeutic measures to accelerate wound healing and reduce the development of refractory ulcers, thus avoiding amputation and reamputation and improving the quality of life of diabetic patients, is a key issue that needs to be addressed in orthopedic clinical practice.

Studies have shown that wound healing is a complex process that can be divided into four stages: coagulation, inflammation, granulation tissue formation, and remodeling or scar formation (Reinke and Sorg, 2012). However, the diabetic wound healing process is severely hampered by a vicious cycle of intertwined factors, such as inadequate tissue nutrients, persistent wound infection, reactive oxygen species (ROS) damage, and abnormal nerve conduction, which ultimately result in a severely hampered wound healing process in diabetic patients (Pawar et al., 2021).

Among the many factors affecting diabetic wound healing, impaired angiogenesis at the wound site, which directly causes a lack of tissue nutrients and severely affects wound regeneration, is the most important cause of failure to heal (Martin, 1997; Zhao et al., 2018; Lin et al., 2020). Since nutrients need to be delivered to the wound site through neovascularization, the amount of neovascularization can directly determine the outcome of diabetic wound healing (Du et al., 2018; Wang et al., 2018). Normal angiogenesis involves processes such as endothelial cell proliferation, migration, and neovascularization, but all of these processes are compromised in diabetic patients (Chao and Cheing, 2009). Although efforts have been made to improve angiogenesis at the wound site, the results of existing therapies, including growth factor therapy, remain unsatisfactory in most cases (Kasiewicz and Whitehead, 2017).

In addition, prolonged exposure of the wound to a pathological inflammatory microenvironment seriously affects the healing process (Wang S et al., 2020). Around chronic diabetic wounds, inflammatory cells are activated and strongly induce the secretion of proinflammatory cytokines (IL-6 and TNF-α), which accumulate in large quantities at the wound site, ultimately leading to severe inflammatory damage (Huang et al., 2019). In addition, glycosylation end products (dAGEs) in diabetic wounds also promote a persistent inflammatory cascade response, which affects the rate of diabetic wound healing (Gao et al., 2021a; Yao et al., 2021).

Chronic bacterial infections also severely affect diabetic wound healing, and the main pathological cause is the colonization of multiple synergistic bacteria, such as *Staphylococcus aureus* and *Escherichia coli*, due to the high glucose levels and low blood supply in the environment around the wound. At the same time, the high glucose levels around chronic wounds in diabetic patients reduce the chemotactic, phagocytic and bactericidal capacity of neutrophils (Zhang C et al., 2019) and significantly reduce the synthesis of antimicrobial components such as ROS and nitric oxide; this causes frequent wound infections (Li X et al., 2018), resulting in the inability to remove colonized bacteria in a timely manner (Liu C et al., 2022). In addition, the lack of antimicrobial components makes it easy for bacteria to form biofilms in a short period of time to resist the host’s immune response and clinical antimicrobial treatments (Sonamuthu et al., 2020), ultimately leading to persistent infections that affect wound healing.

Oxidative stress is another key factor that contributes to delayed diabetic wound healing. Increased production and reduced scavenging of ROS, including superoxide ions, H_2_O_2_ and hydroxyl radicals, around wounds in diabetic patients leads to an imbalance between ROS and endogenous antioxidants in the body (Pasupuleti et al., 2020); this rapid production of excessive ROS leads to mitochondrial dysfunction, which inhibits the binding of vascular endothelial growth factor (VEGF) and its receptors, resulting in impaired endothelial cell chemotaxis and restricted proliferation, impaired angiogenesis (Tatmatsu-Rocha et al., 2018) and ultimately an impaired wound healing process (van Dam, 2002).

In addition, diabetic wound healing is strongly associated with peripheral neuropathy. Segmental axonal demyelination and slow regeneration of injured nerves due to wounds in a state of chronic hyperglycemia and insulin resistance, as well as the development of axonal regeneration defects (Callaghan et al., 2012; Feldman et al., 2017), all lead to increased neurological dysfunction (Zenker et al., 2013). The resulting absence of touch, pain, and/or temperature sensation at the wound (Boulton et al., 1998) is highly likely to cause secondary wound injury and further inhibit wound healing.

Although this review cites angiogenic malformation and inadequate tissue nutrients, persistent wound infection and inflammation, reactive oxygen species (ROS) damage, and abnormal nerve conduction, these are not the only problems faced in the course of chronic diabetic ulcers, nor are there absolute boundaries between these problems, as these factors are often causal or additive. These problems have been selected for discussion of their management in light of their prominence and centrality in the pathology of diabetic chronic ulcers and their clinical value when addressed.

## 2 Current treatment

Considering the above problems of insufficient tissue nutrients, persistent wound infection, ROS damage and abnormal nerve conduction, the current clinical treatments for diabetic wounds mainly include hyperbaric oxygen therapy (HBOT), water-permeable oxygen supplementation, and laser and stem cell therapy. Among these treatments, HBOT is used to treat patients with high concentrations of oxygen at higher than atmospheric pressure, and although this method can enhance the oxygen content of periwound tissues in a short period of time, the therapeutic effect is uncertain, and this approach is accompanied by oxygen radical damage (Guan et al., 2021), pneumatic pressure injury, central nervous system (CNS) damage, and pulmonary oxygen toxicity, all of which seriously limit the clinical application of hyperbaric oxygen (Heyboer et al., 2017). Unlike hyperbaric oxygen, the application of aqueous dissolved oxygen dressings also helps to improve the wound oxygen environment, and its performance is derived from the delivery of dissolved oxygen, which is more than 100 times more efficient than gaseous oxygen in penetrating the skin (Chen et al., 2020), but this adjuvant has a single therapeutic role and cannot achieve effects such as infection control, the promotion of vascular renewal, and nerve repair. In addition, to address the problem of nerve damage in wounds, horizontal laser therapy (LLLT) has emerged (Perez-Favila et al., 2019); this approach uses a laser to promote the rapid regeneration of nerve tissue around the wound while facilitating the production of extracellular matrix components at the wound site (de Alencar Fonseca Santos et al., 2018). However, this method is difficult to develop further due to the high cost of treatment and the limited therapeutic effect (Kaviani et al., 2011).

With the rise of cell therapy in the medical field, scholars have focused on stem cell therapy for chronic diabetic ulcers, and the most widely used mesenchymal stem cells (MSCs) have been shown to increase angiogenesis (Walter et al., 2010), enhance epithelial regeneration, inhibit fibroblast death and extracellular matrix (ECM) loss (Jeon et al., 2010), and regulate inflammation (Luo G et al., 2010). However, studies have shown that diabetic wounds are not conducive to stem cell survival, proliferation, and differentiation and that exogenous stem cells have short survival times, poor stability, and immune rejection in diabetic ulcers (Gouin et al., 2008; Yang et al., 2020). To address these drawbacks, researchers have combined stem cells and hydrogel material to increase the survival time of stem cells and ensure their biological stability, which accelerates wound healing and ensures overall resistance to infection and inflammation due to the presence of hydrogel (Qi et al., 2022). This has gradually opened a new chapter summarized by “the whole is greater than the sum of the parts” for the application of composite hydrogels in chronic diabetic wounds.

## 3 Hydrogel composites

According to their functional classification, the following types of composite hydrogels are currently involved in the field of chronic diabetic wounds (Supplementary Table S1).

### 3.1 Vascular regenerative hydrogels

Among the many factors affecting the healing of chronic diabetic wounds, impaired angiogenesis is very important. To address this problem, researchers have designed different types of proangiogenic hydrogel composites, in which almost all kinds of components have been loaded, such as polymers/polysaccharides/organic chemicals, stem cells/exosomes/progenitor cells, chelating agents/metal ions, plant extracts, proteins (cytokines/peptides/enzymes) and nucleoside products and medicines/drugs. Stem cells/exosomes are the most commonly used.

### 3.2 Anti-inflammatory hydrogels

To address the chronic inflammatory microenvironment of diabetic wounds, researchers have designed various composite hydrogels to achieve anti-inflammatory effects by loading different types of “cargo” to efficiently repair wounds: these anti-inflammatory components mainly include polymers/polysaccharides/organic chemicals, stem cells/exosomes/progenitor cells, chelating agents/metal ions, plant extracts, and medicines/drugs. From these studies, we can see that all these loaded components act on IL-6 and TNF-α, thus promoting the M1 to M2 transition in macrophages (Supplementary Table S2).

### 3.3 Anti-infection hydrogels

Diabetic wounds infected with antibiotic-resistant bacteria are a serious complication of diabetes, and the current misuse of antibiotics is very likely to lead to the development of drug-resistant bacteria near the wound and thus affect wound healing. To address this issue, researchers have designed different types of antimicrobial hydrogel composites, of which there are two main categories. One category exerts antimicrobial effects due to the specific natural polymer compounds in the hydrogel materials. The other category of hydrogel composites is loaded with antimicrobial components to kill colonized bacteria at the wound site and achieve an antimicrobial effect. Among these, there is the very interesting category of loading components that achieve antibacterial effects by reducing the glucose concentration or increasing the concentration of reactive oxygen species since glucose can be oxidized to produce gluconic acid, and H_2_O_2_ can be catalyzed by a cascade reaction and converted to -OH to exert an antibacterial effect.

### 3.4 Antioxidant hydrogels

Oxidative stress is a key factor in delayed diabetic wound healing: studies have shown that the body’s immune cells produce more reactive oxygen species (ROS) under hyperglycemic conditions, and the excessive production of ROS leads to chronic inflammation, which impairs the healing of diabetic skin wounds. Removal of these ROS from wound dressings can provide an effective treatment for chronic wounds, so researchers have developed a series of hydrogel composites to remove ROS. The loading components within the antioxidant hydrogels in this review are basically related to chelating agents and metal ions. In addition to metal ions, there is a special group of ingredients that play a role in antioxidants: plant extracts.

### 3.5 Neural regeneration hydrogels

The absence of tactile sensation, pain and/or temperature caused by the lack of nerve regeneration around the wound during diabetic wound healing is highly likely to cause secondary wound injury and further inhibit wound healing. To address the problem of nerve regeneration in diabetic wounds, researchers have designed two main types of nerve regeneration hydrogel composites, conductive hydrogels containing polypyrrole and composite hydrogels loaded with stem cells/exosomes. The current research on nerve regeneration in chronic diabetic wounds in the presence of hydrogel composites with other materials is insufficient, but the available literature tells us that hydrogel composites can indeed improve and repair nerve damage at the wound site, which provides a basis for future research related to this clinical problem.

### 3.6 Antiglycolytic hydrogels

Although poor angiogenesis, bacterial infection, oxidative stress, and peripheral neuropathy ultimately contribute to chronic wound and ulcer formation in diabetic patients (Dinh et al., 2012), studies have shown that the direct source of these problems is the high-glucose environment surrounding the wound (Malone-Povolny et al., 2019), which induces oxidative stress, leading to mitochondrial damage. Therefore, ameliorating the high-glucose environment is also considered an option to address chronic ulcers in diabetes (Yu et al., 2019; Li S et al., 2021). However, almost all existing composite hydrogels reduce periwound glucose concentrations by involving glucose oxidase (GOx).

**FIGURE 1:**
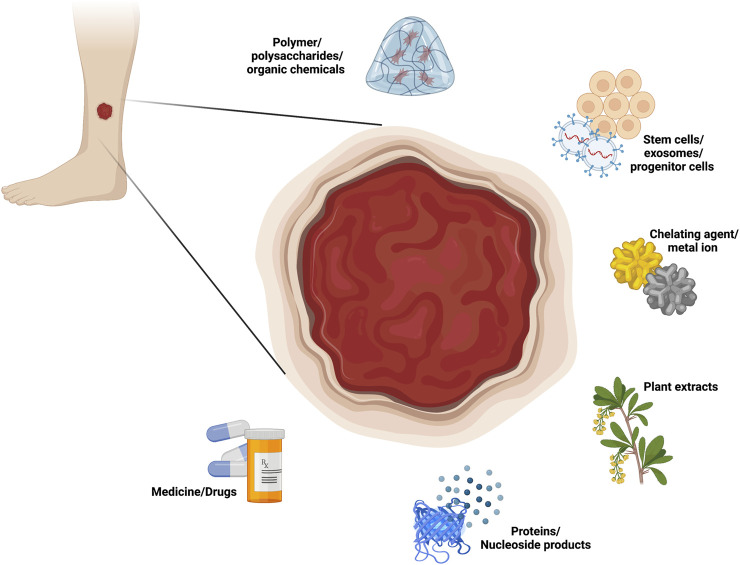
Loaded components in composite hydrogel dressings in diabetic wound healing.

This review focuses on six categories of loaded components in composite hydrogel dressings for use in diabetic wound healing, namely, polymer/polysaccharides/organic chemicals, stem cells/exosomes/progenitor cells, chelating agents/metal ions, plant extracts, proteins (cytokines/peptides/enzymes) and nucleoside products, and medicine/drugs (Figure 1). Each of these loading component types is further divided by function, such as vascular regeneration, anti-inflammatory effects, antibacterial effects, antioxidant effects, nerve regeneration and antiglycolytic effects. Furthermore, this review also discusses a number of components that have not yet been applied but have the potential to be loaded into hydrogels, all of which play roles in the biomedical field and may become important loading components in the future.

## 4 Loaded components

### 4.1 Polymers/polysaccharides/organic chemicals

Hydrogels are composed of 3-dimensional hydrophilic polymer networks. When faced with the problem of chronic diabetic wounds that are difficult to heal, researchers first thought of using the hydrogel itself to achieve results because of all the loading components, the polymer, as the component that directly constitutes the hydrogel, often determines the inherent properties of the hydrogel material and is therefore a research focus (Zhao Y et al., 2020). Below, we list examples of applications of polymers alone that relate to many factors affecting the healing of chronic diabetic wounds. Although some of these hydrogels also achieve some therapeutic effects through their loading components, the ones discussed in this section work solely through the natural properties of the polymer (Figure 1) (Table 1).

**TABLE 1:** Classification of loading components in composite hydrogels in the treatment of chronic diabetic ulcers.

	Hydrogel composites names/Years	PMID/Author	Vascular regeneration/angiogenesis	Anti-inflammatory effect	Antibacterial effect	Antioxidant effect	Nerve regeneration	Antiglycolytic effect
Polymer/Polysaccharide/Organic chemical	OSA-DA-Hydrogel	35101479	Dopamine (DA)					
	2022	Chi, J. et al.						
	GM-OCS-P-Hydrogel	34648694	Oxidized chondroitin sulfate -polypyrrole (OCS-P)					
	2022	Fan, L. et al.						
	P_E_-E_NP_-PCH-Hydrogel	35335913		Chitosan				
	2022	Lee, Y.H. et al.						
	siNP-BG-SA-Hydrogel	35421617		Bioglass (BG)				
	2022	Li, Y. et al.						
	CSGI-Hydrogel	35302563			Carboxymethyl chitosan (CMCS)			
	2022	Ji, S. et al.						
	QCS-TA-Hydrogel	34896467			Quaternized chitosan (QCS)			
	2022	Pan, W. et al.						
	Q-P-D-Hydrogel	35288167			Polyaniline-grafted quaternized chitosan (QCS-P)			
	2022	Wu, C. et al.						
	Ch-AgNPs-Ce-Hydrogel	34774393			Chitosan (CS)			
	2022	Rodriguez-Acosta, H. et al.						
	PUAO-CPO-EXO-Hydrogel	32305816				antioxidant polyurethane (PUAO)		
	2020	Shiekh, P. A. et al.						
	GM-OCS-P-Hydrogel	34648694					Oxidized chondroitin sulfate-polypyrrole (OCS-P)	
	2022	Fan, L. et al.						
	ECH-Hydrogel	33937592					Polypyrrole (PPy)	
	2021	Liu, C. et al.						
	2022	Ma, S. et al.						
	HA-MnO_2_-FGF-2-Exos-Hydrogel	3,4791802	M2-derived Exosomes (M2 Exos)					
	2022	Xiong, Y. et al.						
	EPCs-aFGF@GelMA-Hydrogel	34973992	Endothelial progenitor cells (EPCs)					
	2022	Zhu, H. et al.						
	GelMA-PEGDA-MN-exos-Hydrogel	35305648	HUVECs-exos					
	2022	Yuan, M. et al.						
Stem cells/Exosomes/Progenitor cells	P-Exos-CMC	34706802	Plasma exosomes (P-Exos)					
	2021	Huang, L. et al.						
	PF-127-Hydrogel	34717751		Umbilical cord mesenchymal stem cells (ucMSCs)-derived exosomes				
	2021	Jiao, Y. et al.						
	GG-HA-Hydrogel	28259681 da Silva, L. P. et al.		Human adipose stem cells (hASCs)				
	2017							
	rADSC-MS-Hydrogel	35433645					Rat adipose derived stem cells (rADSCs) rADSCs derived exosomes	
	2022	Shi, M. et al.						
	Chitosan-Silk-Hydrogel	29163228					Human adipose-derived stem cell (hsASCs)	
	2017	Shi, Q. et al.						
	GG-HA-Hydrogel	28259681 da Silva, L. P. et al.					Human adipose-derived stem cell (hsASCs)	
	2017							
Chelating agent/Metal ion	Q-P-D-Hydrogel	35288167	Deferoxamine (DFO)					
	2022	Wu, C. et al.						
	DG-Hydrogel	34879294	Deferoxamine (DFO)					
	2022	Yang, J. et al.						
	SA-DFO-Cu-Hydrogel	35066024	Deferoxamine (DFO), copper nanoparticles (Cu-NPs)					
	2022	Li, S. et al.						
	CSGI-Hydrogel	35302563			Macromolecular optical probe (Ir-fliq-PVP)			
	2022	Ji, S. et al.						
	Ch-AgNPs-Ce-Hydrogel	34774393			Silver nanoparticles (AgNPs)			
	2022	Rodriguez-Acosta, H. et al.						
	nZnO-MIC-Hydrogel	35467827			Zinc oxide nanoparticles (nZnO)			
	2022	Guo, C. et al.						
	DG-Hydrogel	34879294			Zinc ions			
	2022	Yang, J. et al.						
	PAA-CaPs-Nps@GOx-Hydrogel	34308944			Fe_3_O_4_/TiO_2_/Ag_3_PO_4_ nanoparticles			
	2021	Huang, T. et al.						
	Gel-BA-VAN-AgNCs-Hydrogel	34240605			Silver (Ag) nanoclusters			
	2021	Wang, Y. et al.						
	MoS_2_-Au@BSA-Hydrogel	35373522				Bovine serum albumin (BSA) decorated Au		
	2022	Li, Y. et al.						
	PBNPs@PLEL-Hydrogel	35298140				Prussian blue nanoparticles (PBNPs)		
	2022	Xu, Z. et al.						
	HA-MnO_2_-FGF-2-Exos-Hydrogel	3,4791802				Manganese dioxide (MnO_2_) nanoenzymes		
	2022	Xiong, Y. et al.						
	nZnO-MIC-Hydrogel	35467827	Paeoniflorin-encapsulated micelle (MIC)					
	2022	Guo, C. et al.						
	B-G-Hydrogel	35319815		Bletilla Striata polysaccharide (BSP)				
	2022	Liu, J. et al.						
	Ch-AgNPs-Ce-Hydrogel	34774393		Calendula extract				
	2022	Rodriguez-Acosta, H. et al.						
	HA-PF-Hydrogel	34632363		Paeoniflorin (PF)				
	2021	Yang, H. et al.						
	QCS-TA-Hydrogel	34896467			Tannic acid (TA)	Tannic acid (TA)		
	2022	Pan, W. et al.						
	PEG-DA/HA-PBA/MY (PHM)-Hydrogel	35129966				Myricetin (MY)		
	2022	Xu, Z. et al.						
Proteins (Cytokines/Peptides/Enzymes) and Nucleoside products	HA-MnO_2_-FGF-2-Exos-Hydrogel	3,4791802	FGF-2 growth factor					
	2022	Xiong, Y. et al.						
	EPCs-aFGF@GelMA-Hydrogel	34973992	Acid fibroblast growth factor (aFGF)					
	2022	Zhu, H. et al.						
	siNP-BG-SA-Hydrogel	35421617	Small interfering RNA of MMP9 (MMP9-siRNA)					
	2022	Li, Y. et al.						
	P_E_-E_NP_-PCH-Hydrogel	35335913	Epidermal Growth Factor (EGF)					
	2022	Lee, Y.H. et al.						
	G4-Hydrogel	35064773			Guanosine-quadruplex (G4)			
	2022	Li, Y. et al.						
	DP7-ODEX-Hydrogel	34973798			Peptide DP7			
	2022	Wu, S. et al.						
	HP-Hydrogel	29609091					Nerve growth factor (NGF) basic fibroblast growth factor (bFGF)	
	2018	Li, R. et al.						
	DG-Hydrogel	34879294						Glucose oxidase (GOx)
	2022	Yang, J. et al.						
	PAA-CaPs-Nps@GOx-Hydrogel	34308944						Glucose oxidase (GOx)
	2021	Huang, T. et al.						
	IKYLSVN-Hydrogel	31492216						Glucose oxidase (GOx)
	2020	Zhao, Y. et al.						
Medicine/Drugs	GelMA-PEGDA-MN-exos-Hydrogel	35305648	Tazarotene					
	2022	Yuan, M. et al.						
	Gel-BA-VAN-AgNCs-Hydrogel	34240605		Nimesulide (NIM)				
	2021	Wang, Y. et al.						
	Gel-BA-VAN-AgNCs-Hydrogel	34240605			Vancomycin			
	2021	Wang, Y. et al.						
	DP7-ODEX-Hydrogel	34973798			Ceftazidime			
	2022	Wu, S. et al.						

This is a provisional file, not the final typeset article.

#### 4.1.1 Vascular regenerative effects

Fan et al. (2022) developed a GM-OCS-P conductive hydrogel composed of gelatin methylacryl (GM), oxidized chondroitin sulfate (OCS), and OCS-polypyrrole conductive nanoparticles. Conductive polymers (CPs) can strongly promote the viability of vascular smooth muscle cells and human umbilical vein endothelial cells (HUVECs), which in turn can have neovascularization effects. Chi et al. (2022) used sodium-oxidized alginate (OSA) as a matrix material and developed an OSA-DA hydrogel synthesized from dopamine (DA) and OSA. The dopamine (DA) component of this hydrogel significantly promoted the migration and tube formation of human umbilical vein endothelial cells (HUVECs), accelerated angiogenesis, and led to faster healing of chronic diabetic wounds.

#### 4.1.2 Anti-inflammatory effects

Li D et al. (2022) developed an injectable bioglass/sodium alginate (BG/SA) hydrogel loaded with MMP-siNPs to inhibit chronic inflammation in chronic diabetic ulcers since BG products can stimulate macrophages to convert to the M2 phenotype. In addition, Lee and Lin (2022) prepared a chitosan-based heterogeneous composite hydrogel loaded with epidermal growth factor (EGF) and factor (EGF) and polyhexamethylene biguanide (PHMB), named PE-ENP-PCH. The chitosan in this material reduces the serum levels of the proinflammatory factors IL-4, IL-13, and TNF-α while increasing the levels of the anti-inflammatory cytokines IL-10 and TGF-β1, achieving a reduction in the inflammatory response in the wound (Kim, 2018).

#### 4.1.3 Antibacterial effects

Among all hydrogel components, chitosan has been proven to have antimicrobial properties, which are attributed to its chelating capacity and positive charge (Rashki et al., 2021). In a study by Ji et al. (2022), carboxymethyl chitosan (CMCS) and sodium alginate (SA) were cross-linked to form a CSGI hydrogel with a small molecule probe (Ir-fliq) containing an iridium complex with hypoxia-monitoring ability. The hydrogel achieved effective bacterial killing *via* chitosan, and the study showed that the CSGI hydrogel had good antibacterial properties against both Gram-positive and Gram-negative bacteria. In a study by Wu C et al. (2022), the authors constructed a conductive antibacterial hydrogel using quaternized chitosan (QCS) and polyaniline (Pani) after compounding with DFO (named QP-P-D). The positive charge carried on the surface of QCS in this hydrogel can bind to the negative charge of the bacterial cell wall to destroy the cell wall and achieve its bactericidal purpose, and experimental results showed that the bactericidal rate of this conductive hydrogel can reach more than 90%. Similarly, Pan et al. (2022) developed a QCS/TA hydrogel. The quaternized chitosan (QCS) component also has antibacterial ability. In a study by Rodriguez-Acosta et al. (2022), chitosan-based hydrogels loaded with silver nanoparticles and calendula extract (Ch-AgNPs-Ce) were developed, and as in a previous study, these chitosan hydrogels reduced the bacterial load at the wound site.

#### 4.1.4 Antioxidant effects

Studies have been conducted on the use of specific natural polymer compounds to alleviate oxidative stress. For example, Shiekh et al. (2020) developed a PUAO-CPO-EXO hydrogel using the antioxidant polyurethane (PUAO) as a matrix material loaded with adipose-derived stem cell (ADSC)-derived exosomes. In this material, PUAO is a highly porous cryogel with sustained oxygen releasing properties and can reduce oxidative stress.

#### 4.1.5 Nerve regeneration

Fan et al. (2022) developed a GM-OCS-P conductive hydrogel composed of gelatin methylacryl (GM), oxidized chondroitin sulfate (OCS), and OCS-polypyrrole conductive nanoparticles. It can target the PI3K and MEK-ERK pathways by increasing the intracellular Ca^2+^ concentration, which in turn can have neuroprotective and neuroregenerative effects. Similarly, Liu C et al. (2021) developed a biocompatible conductive hydrogel (ECH) that can promote axonal regeneration and myelin regeneration through the MEK/ERK pathway, as well as the proliferation of Schwann cells, which provide metabolic support to regenerating axons. The results demonstrated that this type of hydrogel is an effective treatment for nerve regeneration in diabetic nerve injury. This type of hydrogel exerts neuroregenerative effects by activating specific neuroregenerative pathways by increasing the intracellular Ca^2+^ concentration through hydrogels with ‘conductive properties'.

#### 4.1.6 Summary and future outlook of polymers/polysaccharides/organic chemicals

**FIGURE 2:**
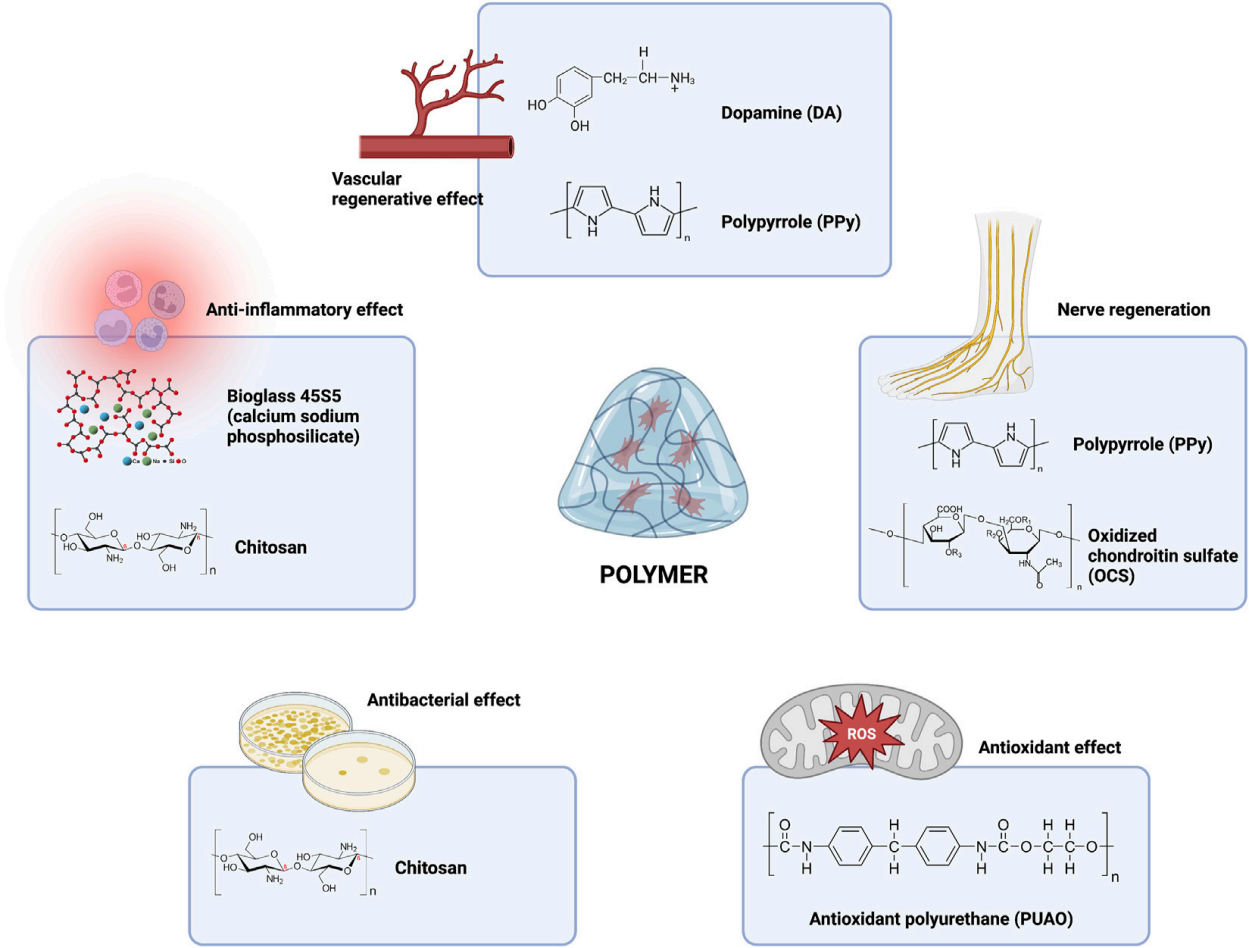
Current and future outlook of polymer components in hydrogel composites in the treatment of chronic diabetic ulcers.

The polymers mentioned in this review mainly include oxidized chondroitin sulfate-polypyrrole (OCS-P) (Fan et al., 2022) and dopamine (DA) (Chi et al., 2022) for vascularization; anti-inflammatory bioglass (BG) (Li S et al., 2022) and chitosan (Lee and Lin, 2022); antibacterial carboxymethyl chitosan (CMCS) (Ji et al., 2022), quaternized chitosan (QCS) (Pan et al., 2022), polyaniline-grafted quaternized chitosan (QCS-P) (Wu J. H et al., 2022), and chitosan (CS) (Rodriguez-Acosta et al., 2022); antioxidant polyurethane (PUAO) (Shiekh et al., 2020); and oxidized chondroitin sulfate-polypyrrole (OCS-P) (Fan et al., 2022) and polypyrrole (PPy) (Liu H et al., 2021) for nerve regeneration. From these studies, we can easily see that components such as chitosan have shown powerful effects in antimicrobial therapy and that polypyrrole, as a conducting polymer, showed nerve regeneration and vascularization effects. Interestingly, polypyrrole was also successfully applied for its conductive properties in the real-time monitoring of glucose levels in DFU (Liu H et al., 2022). Chitosan is one of the most investigated biopolymers for wound healing applications (Miguel et al., 2019) and can effectively inhibit bacterial proliferation, overcoming the deficiency of composite hydrogels in the context of antimicrobial activity (Dai et al., 2011; Paramasivan et al., 2014; Matica et al., 2019; Alven and Aderibigbe, 2020; Baidamshina et al., 2020; Hou et al., 2020; Moeini et al., 2020; Wang Y et al., 2021). To give the hydrogel both antibacterial and vascular and nerve regeneration functions, polypyrrole and chitosan can be used together in future work to achieve multiple effects. In addition, it has been shown that chitosan can promote nerve regeneration by forming special ultrastructures (Liu T et al., 2021). Thus, we speculate that chitosan loading will be an important component of future all-in-one hydrogel composites for diabetic wounds. Of course, in addition to chitosan, common polymers include alginate (Silva et al., 2020), chitin (Park and Kim, 2010), hyaluronic acid (Zhai et al., 2020), pectin (Wu et al., 2020), pullulan (Ma et al., 2018), starch (Waghmare et al., 2018), collagen (Gaspar-Pintiliescu et al., 2019), gelatin (Resmi et al., 2020), *β*-glucan (Singh et al., 2018), cellulose (Portela et al., 2019), and konjac glucomannan (Yang D et al., 2017). All of the above polymers have been used in the field of wound healing, so they should be suitable for use in the treatment of chronic diabetic wounds in the future (Figure 2).

To date, polymers have been widely utilized for chronic diabetic ulcers as delivery systems (Norouzi et al., 2016). As one of the most widely used delivery systems, thermosensitive hydrogels transform instantly into gels at body temperature and can be used to deliver a variety of therapeutic agents (Saravanan et al., 2019; Xiao L et al., 2021). For instance, Li R et al. (2018) used a novel thermosensitive heparin-poloxamer (HP) hydrogel to deliver fibroblast growth factor (bFGF) and nerve growth factor (NGF) to exert neuroregenerative effects in diabetic wounds. In addition, in studies of non-diabetic wounds, Tang et al. (2022) used thermosensitive chitosan-based hydrogels (CHI hydrogels) to deliver miR-432-5p-containing exosomes to promote corneal epithelium. Furthermore, pH-sensitive hydrogels can be designed based on the altered pH environment in diabetic wounds. For instance, Huang T et al. (2021) used a pH-responsive carboxymethylcellulose (CMC) hydrogel to deliver plasma exosomes (P-Exos) and vascular endothelial growth factor (VEGF) to enhance angiogenesis. In addition, multifunctional photoresponsive hydrogels (MPRHs) that integrate the advantages of light and hydrogels are increasingly used in the field of wound repair (Maleki et al., 2021). Various therapeutic agents can be incorporated inside the MPRH to create an outstanding drug delivery system (Li Y et al., 2021). There is no research on the direct application of photosensitive hydrogels to DFU, but this does give us a direction for future research. In addition, as a way to enhance the therapeutic index of hydrogels, nanoparticles have been increasingly combined with polymers to create a hybrid biomaterial system for controlled drug delivery (Gao et al., 2016). For instance, Howard et al. (2009) used chitosan nanoparticles to deliver TNF-α-siRNA to reduce the inflammatory response. Yang et al. (2015) used chitosan nanoparticles to deliver COX-2-siRNA to reduce inflammatory damage in mice. In addition, delivering cargo into cells requires a positively charged delivery system since the biological barriers to drug delivery are commonly negatively charged (Yan et al., 2017). For instance, Lin et al. (2022) used a positively charged polyamidoamine hydrogel to deliver kartogenin (KGN) to chondrocytes. However, Yamada et al. (2021) reported that highly positively charged hydrogels are inherently toxic to cells, which seems to limit their utility. From the above, we can easily see that although current research only utilizes a single-sensitivity hydrogel delivery system to accomplish the delivery of drugs or cellular components, it is possible that in the future, through dual-sensitivity or even multisensitivity hydrogel delivery systems, sequentially delivering several compounds that can act at different stages of wound healing will be possible.

### 4.2 Stem cells/exosomes/progenitor cells

#### 4.2.1 Vascular regenerative effects

Considering the advantages of exosomes for promoting vascular regeneration, researchers have designed a series of exosome-loaded hydrogel composites. Ma et al. (2022) developed porcine small intestinal submucosa-based hydrogel material-peptide-exosomes (SC-Ps-sEVs), which were enriched with Wnt4-loaded ucMSC-derived exosomes to activate the *β*-catenin pathway as a way to promote neovascularization after injury and exhibited an excellent ability to stimulate angiogenesis. Furthermore, the pH-responsive carboxymethylcellulose (CMC) hydrogel loaded with plasma exosomes (P-Exos) that was developed by Huang L et al. (2021) enhanced angiogenesis as well as re-epithelialization by activating the angiogenesis-related pathway mediated by vascular endothelial growth factor (VEGF), thereby promoting diabetic wound healing. Yuan et al. (2022) developed a GelMA/PEGDA MN hydrogel using GelMA and PEGDA as matrix materials and combined this hydrogel with tazarotene and HUVEC exosomes to promote vascular endothelial cell proliferation and accelerate neovascularization, thereby promoting rapid diabetic wound healing. Xiong et al. (2022) developed an HA@MnO_2_/FGF-2/Exos hydrogel in which the hydrogel-loaded M2-derived exosomes (M2 Exos) and FGF-2 synergistically induced angiogenesis, granulation tissue formation, and collagen accumulation, and the study confirmed the significant potential of this hydrogel to promote diabetic skin reconstruction *in vivo*. In addition, loaded vascular-related progenitor cells can be used directly to promote vascular regeneration. Zhu et al. (2022) used methacrylated gelatin as a matrix material and loaded it with endothelial progenitor cells (EPCs) as well as acidic fibroblast growth factor (aFGF), which synergistically upregulated HIF-ɑ levels at the wound site and initiated angiogenesis.

#### 4.2.2 Anti-inflammatory effects

Jiao et al. (2021) designed a PF-127 hydrogel that successfully polarized M1 (proinflammatory) macrophages to M2 (anti-inflammatory and prohealing) macrophages through embedded umbilical cord mesenchymal stem cells (UC-MSCs) and sodium ascorbyl phosphate (SAP), which effectively inhibited the development of further wound inflammation (Louiselle et al., 2021). Similarly, da Silva et al. (2017) developed a composite hydrogel containing human adipose stem cells (hASCs), which modulated the inflammatory response around the nerve fibers that polarized M1 macrophages to M2 *via* loaded hASCs. Here, we can easily see that either polymer- or stem cell-derived exosome components can reduce wound inflammation by inhibiting M1 polarization of macrophages.

#### 4.2.3 Nerve regeneration

In addition, the gelatin microsphere hydrogel developed by Shi et al. (2022) was combined with adipose stem cells (rADSCs) derived from mice, and the results demonstrated that the hydrogel promoted early differentiation of nerve fiber bundles, facilitated neurogenesis, and eventually induced nerve axon growth into the wound bed. Similarly, da Silva et al. (2017) developed a composite hydrogel containing human adipose stem cells (hASCs), which promoted nerve regeneration at the wound site by modulating the inflammatory response around the nerve fibers, thereby promoting diabetic wound healing.

In view of the various problems in the application of stem cells themselves, another approach is to accomplish neural regeneration by loading exosomes in hydrogels derived from different types of stem cells. For example, Shi et al. (2017) designed a chitosan/silk hydrogel compounded with gingival mesenchymal stem cell (GMSC)-derived exosomes, which increased the nerve density at the wound site and promoted the growth of nerve fibers into the wound bed through the effect of nerve growth factor (NGF) release from the exosomes.

#### 4.2.4 Summary and future outlook of stem cells/exosomes/progenitor cells

Exosomes have been a popular research topic in recent years; these bioactive structures can be effectively transported to and received by recipient cells to support cellular functions both *in vitro* and *in vivo*, and they can even confer functions specific to their parent cells (Mittelbrunn and Sanchez-Madrid, 2012) while avoiding the potential safety issues caused by the direct use of cellular products (Shao et al., 2018). The literature suggests that stem cell- and MSC-derived exosome components loaded in composite hydrogels can effectively promote the regeneration of blood vessels and nerves and inhibit the progression of inflammation at the wound site (da Silva et al., 2017; Jiao et al., 2021; Ma et al., 2022; Shi et al., 2022; Zhu et al., 2022). Moreover, it is clear from the cited literature that there has been an increasing number of studies using MSC-derived exosomes to achieve therapeutic effects, since exosomes exert the same therapeutic effect as stem cells in diabetic wound treatment (Shi et al., 2017; Xu et al., 2022a; Xiong et al., 2022). Moreover, the application of stem cell-derived exosomes has solved to some extent the problem that diabetic wounds are unsuitable for stem cell survival, as well as some problems associated with stem cell products. Therefore, we believe that in future studies, all those problems that can be solved by MSCs can be tried by MSC-derived exosomes in the treatment of chronic wounds in diabetes.

However, the current application of MSC-derived exosomes in hydrogels also faces some challenges, since the literature does not cover the application of these components to achieve antibacterial, antioxidant, and antiglycolytic effects. Raghav et al. (2021) reviewed the literature and concluded that MSC-derived exosomes can kill bacteria in wounds by enhancing phagocytosis in macrophages at the wound site, which provides a new idea for the future use of hydrogel-loaded stem cells for wound antimicrobial therapy. In addition, urinary-derived exosomes were reported to possess antimicrobial peptides (AMPs); to contain lysozyme C, dermcidin, mucin-1, calprotectin, and myeloperoxidase; and to have a bactericidal effect (Hiemstra et al., 2014; Alcayaga-Miranda et al., 2017). In the same research, it was concluded that nasal lavage fluid-derived exosomes showed antibacterial effects (Alcayaga-Miranda et al., 2017). In another study, it was reported that exosomes released from the biliary and intestinal epithelium lumens contain AMPs (Hu et al., 2013). In addition, several *in vivo* studies have demonstrated the antibacterial effect of antimicrobial peptides secreted by BM-MSCs (Krasnodembskaya et al., 2010; Harman et al., 2017) and AT-MSCs (Cortes-Araya et al., 2018). In addition, Wang T et al. (2020) found that MSC-derived exosomes can repair oxidative stress-induced skin injury *via* adaptive regulation of the NRF2 defense system. Similar findings were obtained in an antioxidant study on MSCs by Kubben et al. (2016). Thus, MSC-derived exosomes may exert a potential antioxidant effect on DFUs.

**FIGURE 3:**
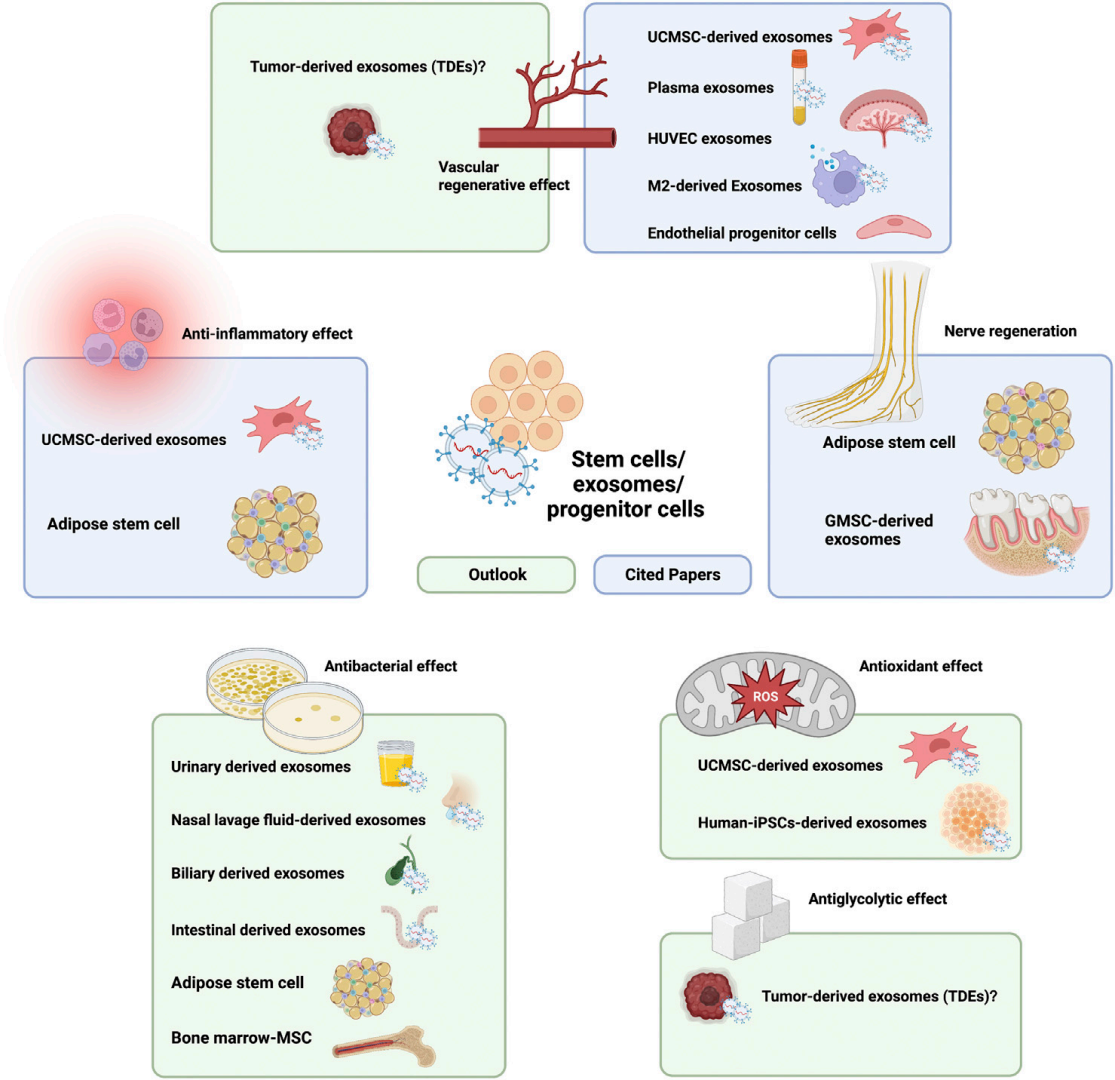
Current and future outlook of stem cell/exosome/progenitor cell components in hydrogel composites in the treatment of chronic diabetic ulcers.

However, we did not find any application for the effect of conventional MSCs or MSC-derived exosomes on local glucose concentrations. One particular type of exosome caught our attention: tumor-derived exosomes (TDEs). Studies have shown that a variety of TDEs are involved in anaerobic glycolysis (Warburg effect) (Cheng et al., 2022). Bladder cancer exosomes promote bladder cancer cell proliferation through miR-4792-mediated enhancement of aerobic glycolysis (also known as the Warburg effect) (Wu S et al., 2022). Exosomes derived from colorectal cancer cells can transfer ciRS-122 across cells and promote aerobic glycolysis in chemosensitive cells (Wang X et al., 2020). Non-small cell lung cancer exosomes can promote lung cancer progression through Circ-MEMO1-mediated aerobic glycolysis (Ding et al., 2020). Nanoblastoma (NB) cell exosomes play a role in chemoresistance *via* aerobic glycolysis (Tan et al., 2022). Exosomes from luminal breast cancer cell exosomes induce an aggressive phenotype through PKM2 phosphorylation-mediated aerobic glycolysis (Kang et al., 2021). All of the above studies demonstrate the potential of TDEs to regulate glucose metabolism, but the application of this particular source of exosomes faces challenges, such as tumorigenicity. However, we believe that these challenges should not prevent the use of TDE in the treatment of DFU in the near future (Figure 3).

### 4.3 Chelating agents/metal ions

#### 4.3.1 Vascular regenerative effects

Deferoxamine (DFO), a chelating agent, has long been used as an antidote for acute iron toxicity, but it has also been shown to promote angiogenesis. DFO stimulates angiogenesis by limiting the degradation of intracellular hypoxia-inducible factor 1-alpha (HIF-1) (Efird et al., 2018). Applying this feature, Wu C et al. (2022) developed a Q-P-D hydrogel consisting of a quaternized chitosan (QCS) and polyethylene glycol (PEG) complex; the addition of DFO promotes the migration of vascular endothelial cells and the expression of HIF-1α and VEGF, synergistically promoting angiogenesis. Similarly, Li W et al. (2022) prepared sodium alginate (SA) hydrogels (SA-DFO/Cu) containing DFO and copper nanoparticles (Cu-NPs) using a Ca^2+^ crosslinking method, and the synergistic effect of DFO and Cu-NPs in this hydrogel enhanced the proliferation and migration of endothelial cells and thus promoted angiogenesis at the wound site to accelerate the healing of chronic diabetic wounds. In addition, in a study by Yang et al. (2022), the investigators developed DG@Gel hydrogels formed by crosslinking metal organic hydrogels (MOGs) and DFO, in which DFO can induce angiogenesis during wound healing in diabetic mice with good biocompatibility and biodegradability. In addition, deferoxamine (DFO) is discussed here in the section on chelating agents/metal ions not only because of its efficient metal binding ability, which allows it to produce a series of wound healing effects, but also because it is almost always found together with metal ions in studies on its use as a hydrogel loading component.

#### 4.3.2 Antibacterial effects

Compared with traditional antibiotics, metal antibacterial agents are more beneficial, as they can overcome bacterial resistance to antibiotics owing to their different antibacterial mechanisms (Xiang et al., 2019). Among the many metal antibacterial agents, zinc ions inhibit the continuous infection of wounds owing to their antibacterial properties (Mao et al., 2017; Zhang Y et al., 2019). Guo et al. (2022) synthesized an injectable ethylenediamine-modified gelatin/oxidized dextran (N-Gel/ODex) hydrogel loaded with zinc oxide nanoparticles (nZnO) and paeoniflorin-encapsulated micelles (MICs). nZnO can stimulate the liberation of Zn^2+^ and the generation of ROS, thereby inducing DNA damage in bacteria. Interestingly, elevated ROS levels contribute to localized angiogenesis. Similarly, Yang et al. (2022) developed DG@Gel hydrogels formed by cross-linking metal organic hydrogels (MOGs) loaded with zinc ions, which can reduce bacteria through the release of zinc ions and the generation of ROS. However, although ROS can be involved in bactericidal activity, there is already increased production and reduced clearance of ROS around diabetic wounds, resulting in a series of problems, such as excessive ROS that can impair mitochondrial function and ultimately cause poor angiogenesis. Thus, it may not be appropriate to promote ROS production for the purpose of bactericidal activity.

With values are similar to those of Zn, silver (Ag) is reported to have extensive antimicrobial properties involving multiple killing mechanisms, such as membrane destruction, ROS production, and DNA damage (Rai et al., 2009). However, the antibacterial activity of Ag is highly dependent on its size; for instance, a smaller nanoparticle size increases the release of Ag atoms and ions and augments antibacterial efficacy. In pursuit of further enhancement of the Ag antimicrobial efficiency, the researchers synthesized ultrasmall Ag nanoclusters. In a study by Rodriguez-Acosta et al. (2022), chitosan-based hydrogels loaded with silver nanoparticles and calendula extract (Ch-AgNPs-Ce) were developed, and as in a previous study, these hydrogels also reduced bacteria counts when loaded with Ag nanoparticles. Wang Y et al. (2021) developed a Gel-BA-VAN-AgNC hydrogel compounded with nimesulide (NIM) and vancomycin-conjugated silver nanoclusters (VAN-AgNCs), which inhibited bactericidal activity through silver nanoclusters. Investigators have also designed a series of different metal antibacterial agents to be loaded into hydrogels. For example, the PAA-CaPs@Nps@GOx hydrogel designed by Huang T et al. (2021) can generate a simple and multifunctional antibacterial platform *via* Fe_3_O_4_/TiO_2_/Ag_3_PO_4_ nanoparticles. In the aforementioned CSGI-hydrogel designed by Ji et al. (2022), a small-molecule probe contained an iridium complex (Ir-fliq). The hydrophilic Ir-fliq has a good ability to generate singlet oxygen and can be used for antimicrobial chemotherapy.

#### 4.3.3 Antioxidant effects

Metal agents act as antioxidants mainly through multienzyme activities. For example, Prussian blue nanoparticles (PBNPs) possess peroxidase (POD), catalase (CAT) and superoxide dismutase (SOD) activities (Zhang et al., 2016). Xu et al. (2022b) developed PBNP@PLEL hydrogels loaded with Prussian blue (ferric ferrocyanide) nanoparticles (PBNPs), which carried PBNPs that were able to resist ROS cytotoxicity and protect mitochondria from oxidative stress-related damage in an oxidative stress environment, thereby improving wound healing.

Similarly, gold nanoparticles (Au NPs) can mimic natural GOx to catalyze the oxidation of glucose into H_2_O_2_ and gluconic acid. The ultimate effect is to scavenge oxygen free radicals (Hu et al., 2020). In a study by Li et al. (2022a), the authors designed injectable hydrogels loaded with defect-rich molybdenum disulfide (MoS_2_) nanosheets and bovine serum albumin-modified gold nanoparticles (Au@BSA). BSA decoration and MoS_2_ in the hydrogel (MoS_2_-Au@BSA-hydrogel) decreased the particle size of Au, resulting in enhanced GOx-like activity, superoxide dismutase (SOD)-like activity, and catalase (CAT)-like activity, which presented excellent antioxidant properties and -OH scavenging ability. Furthermore, the hydrogel composites constructed an antioxidant defense system through peroxidase (POD) and glucose oxidase (GOx) and accelerated the consumption of glucose around the diabetic wound. However, Au NPs have limitations in that they do not have a uniform ultrasmall particle size to enhance activity (Luo W et al., 2010).

It was recently shown that manganese dioxide (MnO_2_) can induce the decomposition of endogenous ROS into oxygen and thus effectively ameliorate oxidative stress (Bao et al., 2018; He et al., 2018). Xiong et al. (2022) developed an HA@MnO_2_/FGF-2/Exos hydrogel that could promote the degradation of ROS using MnO_2_ nanoenzymes embedded in the hydrogel, effectively eliminating H_2_O_2_-induced ROS, mitigating oxidative stress-induced damage, and protecting wounds from the oxidative microenvironment.

#### 4.3.4 Summary and future outlook of chelating agents/metal ions

**FIGURE 4:**
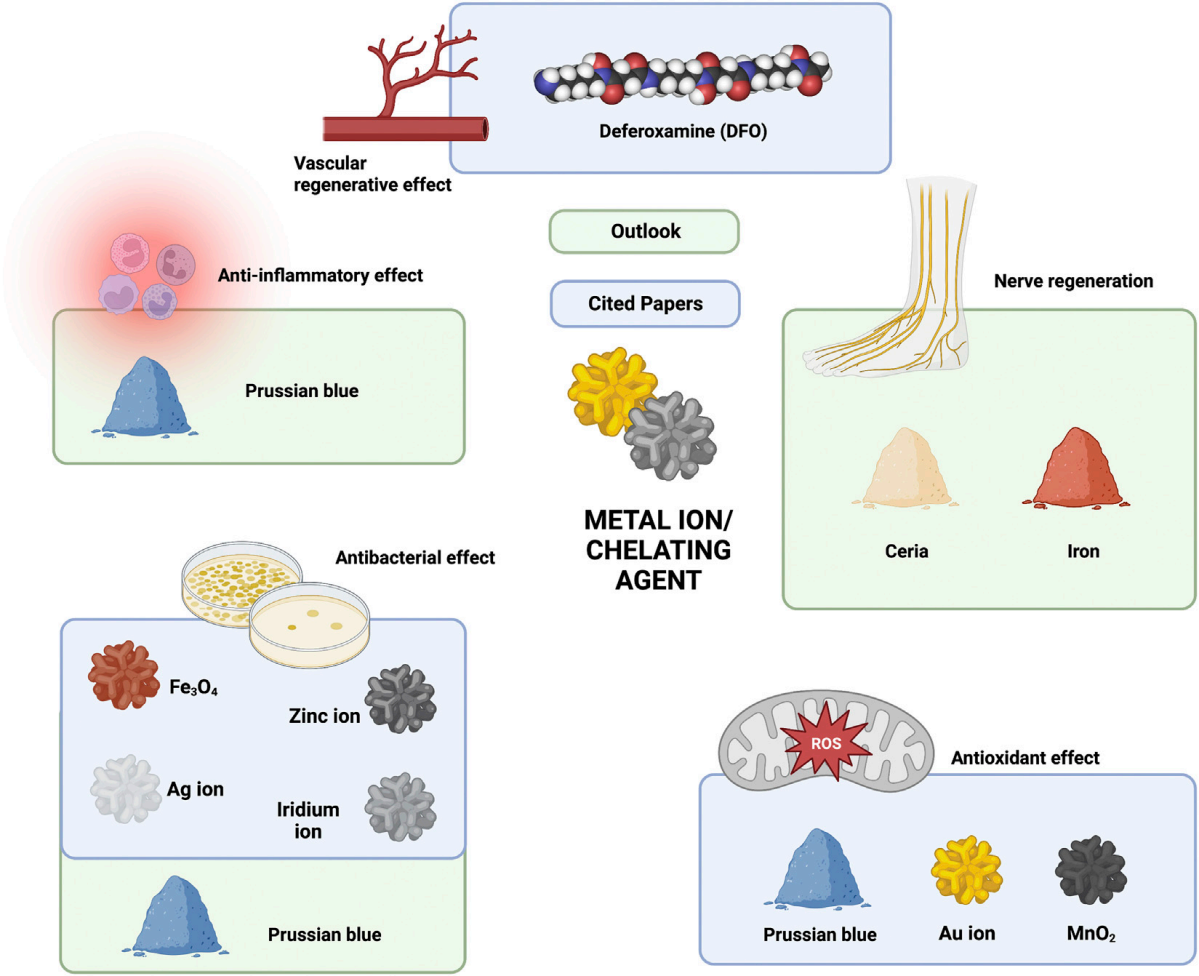
Current and future outlook of chelating agent/metal ion components in hydrogel composites in the treatment of chronic diabetic ulcers.

In studies on the application of hydrogels in diabetic chronic ulcers, the chelating agent/metal ion component was mainly involved in vascular regeneration, antibacterial effects, and antioxidant effects. Deferoxamine (DFO) is used to promote angiogenesis (Li et al., 2022b; Wu J. H et al., 2022; Yang et al., 2022), Ag/Zn/Fe is used as an antibacterial agent (Huang L et al., 2021), and Prussian blue, Au and Mn are involved in the scavenging of ROS (Li et al., 2022b; Xu et al., 2022b; Xiong et al., 2022). Interestingly, Zhang et al. (2022) and Li et al. (2022a) found that Prussian blue also has excellent anti-inflammatory and antibacterial effects due to the generation of ROS *via* the Fenton reaction. This study also provides a theoretical basis for future antimicrobial applications of Prussian blue in chronic diabetic wounds. The simultaneous anti-inflammatory, antioxidant, and antibacterial effects make Prussian blue an ideal loading component for future “all-in-one” applications. In addition, it has been shown that Ceria nanoparticles can ameliorate neurological damage by promoting remyelination after intracerebral hemorrhage (Zheng et al., 2021). Such findings suggest that the Ceria ion should be regarded as a functional unit in nerve regeneration in DFU. Another study demonstrated that iron chelation plays a critical role in promoting neurite growth in nerve regeneration (Mietto et al., 2021). Although there are no studies on the use of chelating agents/metal ions as loading components in hydrogels for the treatment of DFU, the beneficial effects of metals on nerves suggested by the above studies still give us hope that composite hydrogels loaded with chelating agents/metal ions are a promising future approach in the field of nerve regeneration (Figure 4).

### 4.4 Plant extracts

#### 4.4.1 Vascular regeneration/angiogenesis

It was recently shown that paeoniflorin, a chemical compound derived from the herb *Paeonia lactiflora,* has the potential to improve angiogenesis (Yang et al., 2021). This special feature is being applied to the development of hydrogel composites. Guo et al. (2022) synthesized an injectable ethylenediamine-modified gelatin/oxidized dextran (N-Gel/ODex) hydrogel loaded with zinc oxide nanoparticles (nZnO) and paeoniflorin-encapsulated micelles (MICs). This hydrogel can rapidly release *paeoniflorin* (PF) to promote angiogenesis triggered by elevated ROS. From the available literature, it is easy to see that the hydrogel is one of the few that promotes angiogenesis through plant extracts.

#### 4.4.2 Anti-inflammatory effects

Plant extracts have also been used in the treatment of chronic diabetic ulcers due to their natural anti-inflammatory properties (Rogerio et al., 2010). In addition, Liu C et al. (2022) designed a double cross-linked bioactive B-G hydrogel, and the addition of *Bletilla striata* polysaccharide (BSP) to the B-G hydrogel caused a significant decrease in the expression of the proinflammatory factors TNF-α and IL-6 in the wound, in addition to promoting the expression of the anti-inflammatory factors IL-10 and TGF-β. The hydrogel showed significant collagen deposition after application in chronic wounds, demonstrating a strong chronic wound management ability. In a study by Rodriguez-Acosta et al. (2022), chitosan-based hydrogels loaded with silver nanoparticles and calendula extract (Ch-AgNPs-Ce) were developed, and similar to a previous study, the calendula extracts in these hydrogel composites inhibited the development of further inflammation. Yang et al. (2021) developed an HA-PF hydrogel system loaded with *paeoniflorin* (PF), which significantly increased the levels of Arg-1, IL-10 and TGF-β (three functional markers associated with M2 macrophages) *via* PF, mediating the transition of macrophages from the M1 to M2 phenotype and thus inhibiting inflammation in diabetic wounds.

#### 4.4.3 Antibacterial and antioxidant effects

Pan et al. (2022) developed a QCS/TA hydrogel by adding tannic acid (TA) on top of QCS, in which tannic acid can effectively destroy the biological structure of bacteria at the wound site with excellent bactericidal ability. In addition, as a small natural molecule with a high phenolic hydroxyl content, tannic acid has excellent ROS scavenging capacity. A study by Xu et al. (2022a) used glucose-sensitive phenylboronic acid (PBA) to modify hyaluronic acid (HA) chains by one-step synthesis, followed by incorporation into a polyethylene glycol diacrylate (PEG-DA) hydrogel matrix to obtain a novel hybrid hydrogel (PEG-DA/HA-PBA), which was loaded with myricetin (MY) with strong antioxidant activity to effectively scavenge ROS and modify the oxidative wound microenvironment.

#### 4.4.4 Summary and future outlook of plant extracts

As an important type of component of hydrogel composites, plant extracts are mainly involved in four functions: vascular and nerve regeneration, anti-inflammatory effects, and antioxidant effects. Some plant extracts have more than one effect; for example, tannic acid (TA) can both promote ROS scavenging and effectively destroy biological structures (Yang et al., 2021; Xu et al., 2022a; Liu J et al., 2022). In addition, paeoniflorin (PF) can promote angiogenesis, while inhibiting PF can promote angiogenesis. These plant extracts with multiple effects also guide future development. In review, it is easy to see that the plant extracts currently used in the treatment of chronic diabetic ulcers barely exhibit antimicrobial effects. However, from the literature, we found that tannic acid (TA) (Chung et al., 1998; Wang et al., 2022), paeoniflorin (PF) (Bai et al., 2021), calendula extract (Abudunia et al., 2017), and myricetin (MY) (Liu C et al., 2021) also have antibacterial effects. In addition to existing plant extracts that are already used in hydrogels, studies have shown that plant extracts, such as extracts from *Curcuma longa* (curcumin) (Saranya et al., 2018; Jirofti et al., 2021), *Rubus chamaemorus* leaves (Thiem and Goslinska, 2004), and *peony flowers* (Vitex pinnata) (Shafie et al., 2020), can exhibit powerful antibacterial abilities when applied to wounds. Since these components have already been loaded into the corresponding hydrogels, only their anti-infection ability remains to be verified, and there is no difficulty in loading them.

In addition, the cited literature reports few plant extracts applied to nerve regeneration, but in fact, current research shows that many herbs have the function of promoting Schwann cell proliferation as well as nerve regeneration, such as *Cervus elaphus sibericus* (CES) (Hong et al., 2021b), *Alpinia oxyphylla* (Ju et al., 2015; Chang et al., 2017; Duan et al., 2020), *Centella asiatica* (Soumyanath et al., 2005; Gregory et al., 2021), *Achyranthes bidentata* polypeptides (ABPP) (Cheng et al., 2014), emodin (Leung et al., 2020), *Inula britannica* var. *chinensis* (IBC) (Hong et al., 2021a), and *Ramulus cinnamomi* (Yang H et al., 2017).

**FIGURE 5:**
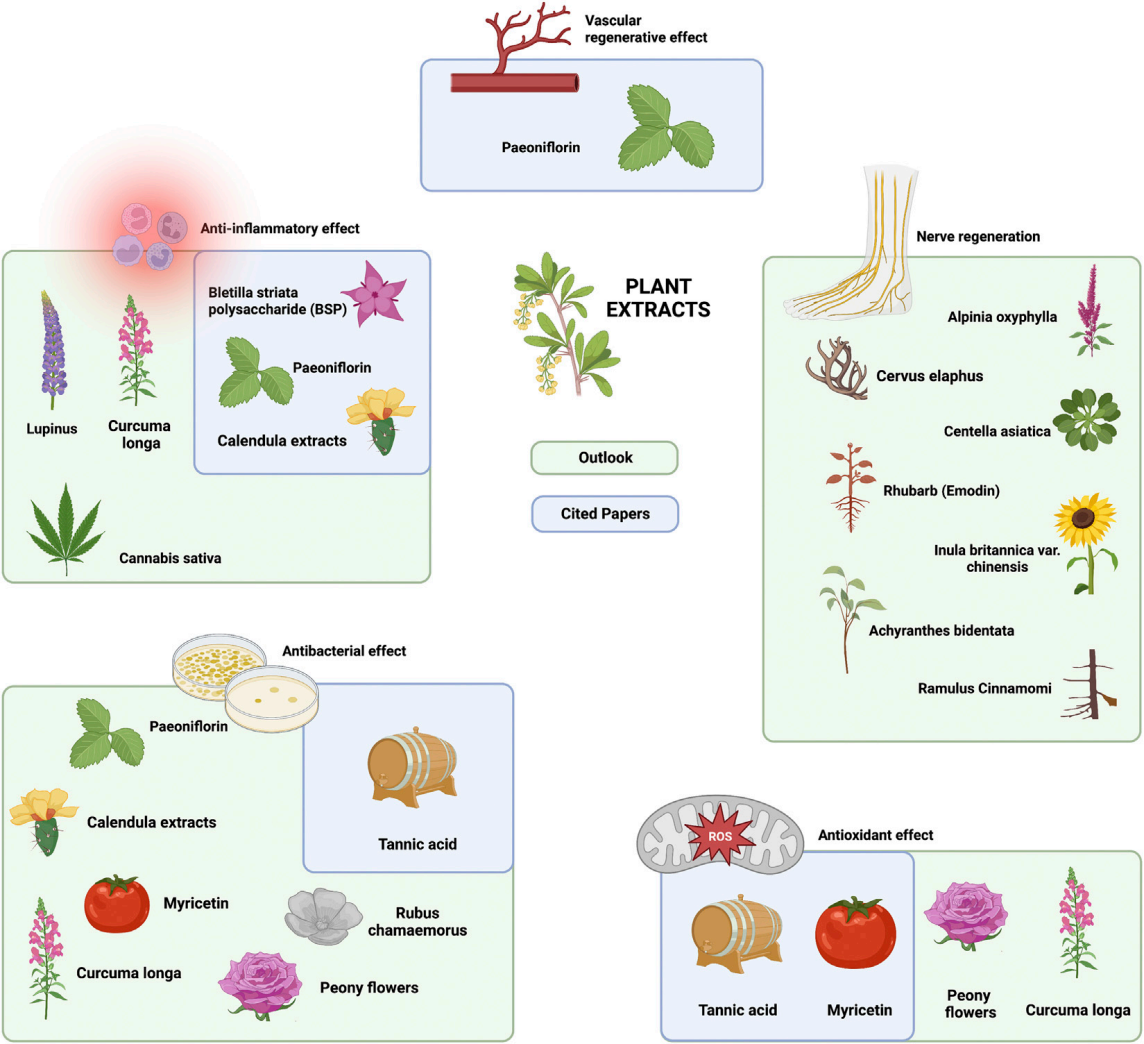
Current and future outlook of plant extracts in hydrogel composites in the treatment of chronic diabetic ulcers.

Moreover, *Cannabis sativa* (Sangiovanni et al., 2019) and lupinol (Saleem, 2009) can suppress wound inflammation. Among these extracts, *Curcuma longa* (containing curcumin) and peony flowers (*Vitex pinnata*) provide additional antioxidant activity against wounds. Interestingly, *Curcuma longa* (containing curcumin) is also effective in achieving anti-inflammatory effects (Saranya et al., 2018; Jirofti et al., 2021) on wounds, making it a very good ‘all-in-one’ loading component. RCT studies have been conducted to validate the oral intake of *Curcuma longa* (containing curcumin) and provide safety information for its future application in composite hydrogels for the treatment of diabetic chronic ulcers (Mokhtari et al., 2021). Furthermore, studies have shown that individual herbs are insufficient to achieve a desired therapeutic effect, and multiple herb compositions in a particular ratio will give a better therapeutic effect (Dev et al., 2019). *Curcuma longa* (containing curcumin) can be considered in combination with the previously mentioned angiogenic plant extract paeoniflorin (PF). In the future, all these extracts could be used for hydrogel loading, which could directly influence wound healing *via* inflammation inhibition, the killing of colonized bacteria and ROS clearance (Biswas et al., 2017) (Figure 5).

### 4.5 Cytokines/proteins/peptides/enzymes/nucleoside products

#### 4.5.1 Vascular regeneration/angiogenesis

Fibroblast growth factor (FGF) is a family of cell signaling proteins that can mediate various processes, such as angiogenesis, to enhance wound healing effects (Zhang et al., 2018). As a powerful angiogenesis factor, it has been loaded into composite hydrogels by a number of researchers. In the abovementioned HA@MnO_2_/FGF-2/Exos hydrogel developed by Xiong et al. (2022), FGF-2 and M2-derived Exosomes (M2 Exos) synergistically induced angiogenesis. The study confirmed the significant potential of this hydrogel to promote diabetic skin reconstruction *in vivo*. Similarly, the previously mentioned EPC-aFGF@GelMA-Hydrogel was designed by Zhu et al. (2022) in which loaded acidic fibroblast growth factor (aFGF) and endothelial progenitor cells (EPCs) synergistically initiated angiogenesis. In addition, Lee and Lin (2022) prepared a chitosan-based heterogeneous composite hydrogel loaded with epidermal growth factor (EGF) to induce angiogenesis. It is interesting to note that according to the above literature, signaling proteins such as FGF are often used in conjunction with stem cells/exosomes. Small interfering RNA (siRNA) technology has also been used in hydrogel composites due to its negligible side effects and promising angiogenic effect. In the abovementioned injectable siNP-BG-SA-Hydrogel developed by Li et al. (2022c) was loaded with small interfering RNA of MMP-9 (MMP-9-siRNA), and the MMP-9-siNPs could effectively downregulate the expression and activity of MMP-9 and promote angiogenesis.

#### 4.5.2 Antibacterial effects

Antimicrobial peptides, a new technology of high interest, are also used in the antimicrobial treatment of chronic diabetic wounds due to their extremely high antimicrobial efficiency. Wu S et al. (2022) developed a DP7-ODEX (DP7 antimicrobial peptide-oxidized dextran) hydrogel to target multidrug-resistant bacteria. In this hydrogel, the DP7 antimicrobial peptide synergized with ceftazidime loaded in the hydrogel to achieve effective killing of colonized bacteria in the wound. In molecular biology, G-quadruplex secondary structures (G_4_) are formed in nucleic acids by sequences that are rich in guanine, and studies have shown that they can achieve a bactericidal effect by converting the H_2_O_2_ produced by glucose oxidation to -OH with the help of heme bound to G-tetraspanin for sterilization purposes. The investigators applied this property to the antimicrobial treatment of chronic diabetic wounds. A study by Li D et al. (2022) involved the development of a guanosine-tetramer (G _4_)-hydrogel. It was shown that this method achieves efficient inactivation of drug-resistant bacteria while avoiding the use of antibiotic-like drugs, providing a very good approach for the future treatment of diabetic wounds.

#### 4.5.3 Nerve regeneration

Among the many growth factors (GFs) that show promise for nerve regeneration, nerve growth factor (NGF) (Zhao et al., 2016; Indo, 2018) and basic fibroblast growth factor (bFGF) (Dong et al., 2019) are the most widely studied. Therefore, these GFs are also used in the treatment of chronic diabetic wounds. A novel thermosensitive heparin-poloxamer (HP) hydrogel developed by Li R et al. (2018) can be codelivered with basic fibroblast growth factor (bFGF) and nerve growth factor (NGF) to exert neuroregenerative effects by activating the specific neuroregenerative pathways PI3K/Akt, JAK/STAT3 and MAPK/ERK. Moreover, although growth factors are potent stimulators of nerve regeneration, cytokines have rarely been applied to hydrogels, primarily due to their rapid degradation and redistribution (Tsai et al., 2003). Therefore, it is critical to use a delivery vehicle that not only maintains GF bioactivity and bioavailability but also controls their spatiotemporal release.

#### 4.5.4 Antiglycolytic effects

Glucose oxidase is an oxidoreductase that catalyzes the oxidation of glucose to hydrogen peroxide. Because of its ability to lower the concentration of glucose, many existing studies have focused on glucose oxidase (GOx). For example, the PAA-CaPs@Nps@GOx hydrogel designed by Huang T et al. (2021) can prevent GOx from being degraded by the body and thus maintain a high level of GOx activity. This allows GOx to convert glucose around the wound into gluconolactone, effectively alleviating the high-glucose environment of the wound. In a study by Yang et al. (2022), the investigators developed DG@Gel hydrogels by cross-linking metal organic hydrogels (MOGs) and DFO, which also reduced the glucose concentration in the wound microenvironment by decomposing the excess glucose at the wound into H_2_O_2_ and glucuronic acid through loaded GOx. Similarly, Zhao Y et al. (2020) designed an IKYLSVN (Ac-Ile-Lys-Tyr-Leu-Ser-Val-Asn-nh2 heptapeptide cross-linked) hydrogel containing GOx to ameliorate the high-glucose environment in wounds by converting glucose to H_2_O_2_ by loading packaged GOx to promote wound healing.

#### 4.5.5 Summary and future outlook of proteins (cytokines/peptides/enzymes) and nucleoside products

In addition to plant extracts, researchers have accelerated diabetic wound angiogenesis by loading cytokines such as FGF (Chi et al., 2022; Zhu et al., 2022). As the effects of various cytokines are reflected in all stages of wound healing (Su et al., 2019), there are a large number of cytokines that can directly affect wound healing, in addition to the existing cytokines that have already been loaded in hydrogels previously mentioned. For example, CC chemokines, CXC chemokines, CX3C chemokines and C chemokines can promote the regeneration of blood vessels around wounds (Ridiandries et al., 2018; Raziyeva et al., 2021). In contrast, there are cytokines that are detrimental to the healing of chronic wounds, and inhibitors of these cytokines can be used to promote the healing of diabetic chronic wounds. Recently, small interfering RNA (siRNA) technology has become a promising approach for treating chronic diabetic wounds, as it can destroy target mRNAs and silence target RNA without causing any side effects. In a previously mentioned study (Li S et al., 2022), researchers used siRNA-MMP-9 to promote angiogenesis in chronic diabetic ulcers, but this is not an isolated case. For instance, Castleberry et al. (2016) applied MMP9-siRNA to treat diabetic chronic wounds. In addition, there are a large number of cytokines that can regulate the progression of inflammation in cellular wounds, for example, proinflammatory cytokines, mainly tumor necrosis factor (TNF), interleukin 6 (IL-6), interleukin 1 (IL-1) and interferon (IFN) (Ridiandries et al., 2018; Nosenko et al., 2019). However, siRNA cannot be used alone because it is difficult for siRNA alone to enter cells, as it is negatively charged and is repelled from the cell surface (Mao et al., 2010). Thus, delivering siRNA into cells requires a positively charged delivery system, and accordingly, a new polysaccharide is needed. For instance, Howard et al. (2009) used TNF-α-siRNA-chitosan nanoparticles to reduce the inflammatory response. Yang et al. (2015) used COX-2-siRNA-chitosan nanoparticles to reduce kidney damage in mice. Although the above studies were not applied to wound healing, they provide theoretical information for future applications in diabetic chronic ulcers. In light of the diverse effects of different cytokine classes, it is possible to create “all-in-one” composite hydrogels for diabetic wounds in the future by combining these factors in hydrogel carriers.

In contrast to the body of research on vascular regenerative and neuroregenerative hydrogels, there are a limited number of studies investigating the application of composite hydrogel-loaded cytokines to nerve regeneration in chronic diabetic wounds. However, applications in nerve regeneration are not uncommon. For example, IL-10-releasing hydrogels can promote the neural differentiation of stem cells (Shen et al., 2022), and hydrogels loaded with brain-derived neurotrophic factor (BDNF) can promote the regeneration of neural Schwann cells (Rao et al., 2020). These cytokines already loaded into hydrogels can be tried in the future for DFU treatment since the focus is not on the cytokines themselves but on preventing the rapid degradation and redistribution of cytokines through the hydrogel.

**FIGURE 6:**
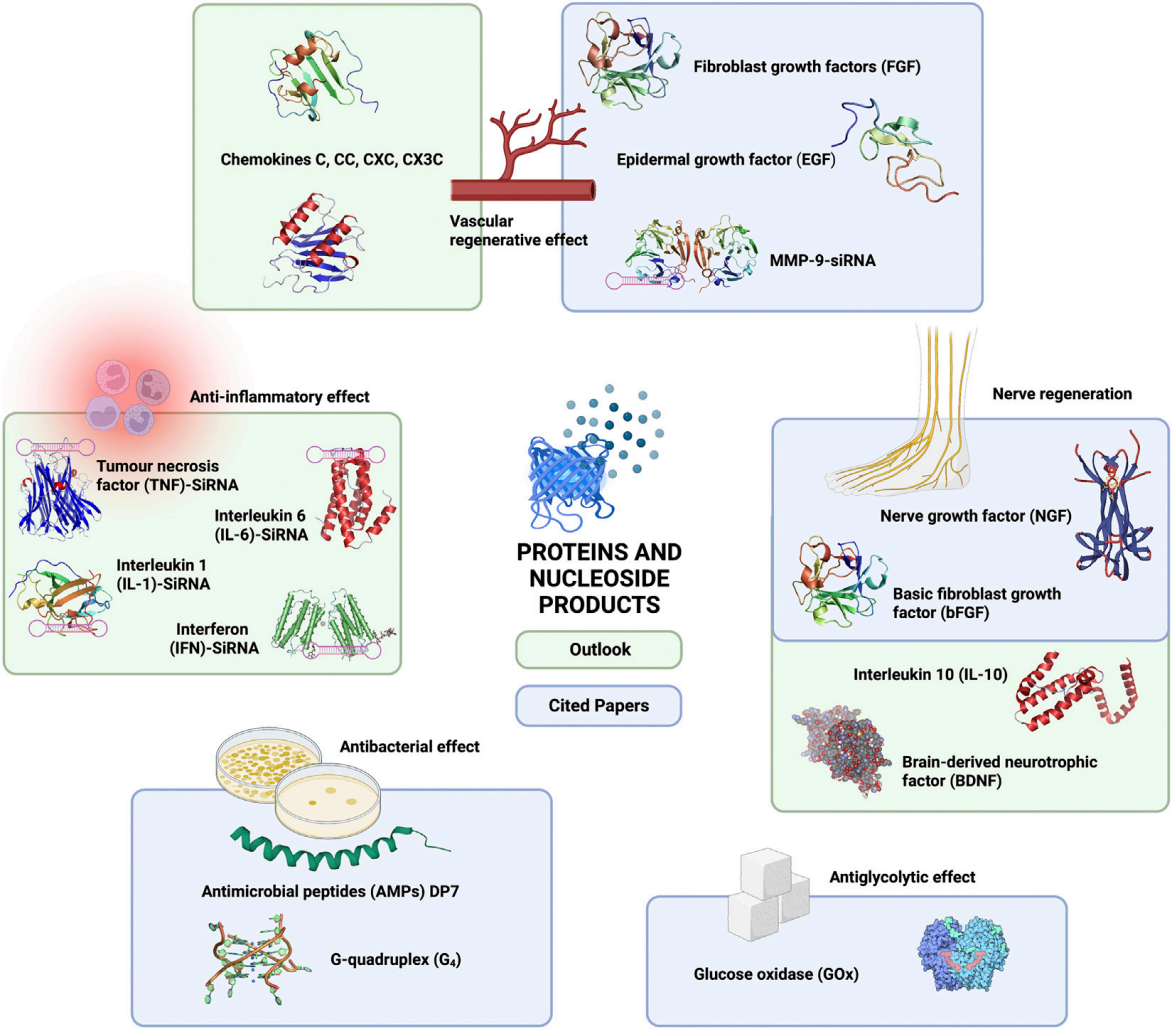
Current and future outlook of proteins (cytokines/peptides/enzymes) and nucleoside products in hydrogel composites in the treatment of chronic diabetic ulcers.

Among the many components loaded into hydrogels, bioenzymes are a unique category and are the only factors in the articles cited that directly reduce the glucose concentration in wounds (glucose oxidase (GOx)) (Zhao C et al., 2020; Huang L et al., 2021b; Yang et al., 2022). In addition to the bioenzymes previously loaded in hydrogels, many types of bioenzymes have been applied in wound therapy, such as trypsin, tyrosine kinase, and matrix metalloproteinases (MMPs), which can effectively inhibit inflammation as well as ROS production (Xue et al., 2006; Shah et al., 2014; Gao et al., 2015; Shah and Mital, 2018). Furthermore, related studies have shown that bioenzymes, such as lysozyme, artificial enzymes, and nanoenzymes exhibiting superior antimicrobial properties, can also remove colonized bacteria from wound surfaces (Chen et al., 2018; Altin et al., 2021; Lin et al., 2021; Xiao Y et al., 2021). Although we are currently unable to perform all the functions of promoting the healing of diabetic chronic ulcers with a single type of enzyme, it can be seen that in the future, the loading of different types of bioenzymes can achieve antiglycolytic, anti-inflammatory, antibacterial and antioxidative effects on wounds. This suggests that bioenzymes also have the ability to support the construction of “all-in-one” composite hydrogels in the future. Of course, the hydrogel composites used in the treatment of chronic diabetic ulcers are not always loaded with the single components mentioned in this study, but there are also mixed components such as platelet-rich plasma (PRP) (Qian et al., 2020). However, since the therapeutic aspects of PRP are still controversial, they are beyond the scope of this review (Figure 6).

### 4.6 Medicines/drugs

The application of drug-loaded hydrogels for the treatment of chronic diabetic wounds has also grown considerably in recent years, especially in the case of certain topical drug components for the treatment of dermatological conditions. For instance, the previously mentioned GelMA/PEGDA MN hydrogel was developed by Yuan et al. (2022) using tazarotene to promote vascular endothelial cell proliferation and accelerate neovascularization. In addition, some non-steroidal anti-inflammatory drugs (NSAIDs) with analgesic and fever-reducing properties are also loaded in hydrogels. The abovementioned Gel-BA-VAN-AgNC hydrogel was developed by Wang C. H et al. (2021) using nimesulide (NIM) to inhibit chronic inflammation in chronic diabetic ulcers. In addition, it uses vancomycin to inhibit bactericidal activity. Although there is a wide range of anti-inflammatory drugs, few have been directly loaded in hydrogels for chronic diabetic ulcers. The success of the above studies in applying commercialized drugs in composite hydrogels indicates promise based on the good biocompatibility of hydrogels, which greatly enhances the range of hydrogel loadings while significantly reducing the development cycle of loadings. Inevitably, however, the role of the hydrogel itself is weakened in this process.

## 5 Conclusion

In this review article, we describe how different types of loading components of current composite hydrogel materials are used in various aspects of diabetic chronic ulcer therapy, such as polymers/polysaccharides/organic chemicals, stem cells/exosomes/progenitor cells, chelating agents/metal ions, plant extracts, proteins (cytokines/peptides/enzymes) and nucleoside products, and medicines/drugs. We also discuss a number of components that have not yet been applied but have the potential to be loaded into hydrogels, all of which play a role in the biomedical field and may become important loading components for DFU in the future. This provides a “loading component shelf” for researchers of composite hydrogels. In addition, we envision the construction of “all-in-one” hydrogels. The combination of different loading components to produce composite hydrogels with specific biomedical properties holds promise for the development of new biomaterials for the treatment of DFU.
