# Supplementary material for: Current and future outlook of loaded components in hydrogel composites for the treatment of chronic diabetic ulcers
Source: Front Bioeng Biotechnol. 2023 Feb 13;11:1077490. doi: 10.3389/fbioe.2023.1077490 (PMC9968980; doi:10.3389/fbioe.2023.1077490)
Supplement: Supplementary file 1 [file Table1.docx]

**Supplementary Table 1. Overview of loading components in composite hydrogels and their functional process**

|  | Hydrogel Composites Names | Years | Authors | Effective components | The effect of process | PMID |
| --- | --- | --- | --- | --- | --- | --- |
| vascular regeneration/angiogenesis | OSA-DA-Hydrogel | 2022 | Chi, J. et al. | Dopamine (DA) | HUVECs ↑→ angiogenesis↑ | 35101479 |
|  | Q-P-D-Hydrogel | 2022 | Wu, C. et al. | Deferoxamine (DFO) | HIF-1α↑，VEGF ↑，HUVECs↑→ angiogenesis↑ | 35288167 |
|  | SA-DFO-Cu-Hydrogel | 2022 | Li, S. et al | Deferoxamine (DFO), copper nanoparticles (Cu-NPs) | HUVECs ↑→ angiogenesis↑ | 35066024 |
|  | SC-Ps-sEVs-Hydrogel | 2022 | Ma, S. et al. | Umbilical cord mesenchymal stem cells (ucMSCs)-derived exosomes | VEGF ↑→ angiogenesis↑ | 35156747 |
|  | HA-MnO_2_-FGF-2-Exos-Hydrogel | 2022 | Xiong, Y. et al | M2-derived Exosomes (M2 Exos), FGF-2 growth factor | FGF-2 growth factor → miR-223↑ → angiogenesis↑ | 34791802 |
|  | GelMA-PEGDA-MN-Hydrogel | 2022 | Yuan, M. et al | Tazarotene, HUVECs-exos | VEGF↑ → angiogenesis↑ | 35305648 |
|  | siNP-BG-SA-Hydrogel | 2022 | Li, Y. et al | Small interfering RNA of MMP9 (MMP9-siRNA) | MMP9↓ → angiogenesis↑ | 35421617 |
|  | GM-OCS-P-Hydrogel | 2022 | Fan, L. et al | Oxidized chondroitin sulfate (OCS), OCS-polypyrrole (OCS-P) | conductive microenvironment → angiogenesis↑ | 34648694 |
|  | EPCs-aFGF@GelMA-Hydrogel | 2022 | Zhu, H. et al | Endothelial progenitor cells (EPCs), acid fibroblast growth factor (aFGF) | HIF-ɑ↑ → angiogenesis↑ | 34973992 |
|  | nZnO-MIC-Hydrogel | 2022 | Guo, C. et al | Paeoniflorin-encapsulated micelle (MIC) | MIC →low-pH- and ROS-stimulated angiogenic activity | 35467827 |
|  | DG-Hydrogel | 2022 | Yang, J. et al. | Deferoxamine (DFO) | HIF-1α↑，VEGF ↑，HUVECs↑→ angiogenesis↑ | 34879294 |
|  | P_E_-E_NP_-PCH-Hydrogel | 2022 | Lee, Y.H. et al | Epidermal Growth Factor (EGF) | EGF↑→ angiogenesis↑ | 35335913 |
|  | P-Exos-CMC | 2021 | Huang, L. et al | Plasma exosomes (P-Exos) | VEGF ↑ → angiogenesis↑ | 34706802 |
|  |  |  |  |  |  |  |
| anti-inflammatory effect | P_E_-E_NP_-PCH-Hydrogel | 2022 | Lee, Y.H. et al | Chitosan | (pro-inflammation) TNF-α↓, IL-6 | 35335913 |
|  | siNP-BG-SA-Hydrogel | 2022 | Li, Y. et al | Bioglass (BG) | M1(pro-inflammation) → M2 (anti-inflammation) → IL-6↓, IL-1β↓, IL-10↑, TGF-β↑ | 35421617 |
|  | B-G-Hydrogel | 2022 | Liu, J. et al | Bletilla Striata polysaccharide (BSP) | (pro-inflammation) TNF-α↓, IL-6↓, (anti-inflammation) IL-10↑, TGF-β↑ | 35319815 |
|  | Ch-AgNPs-Ce-Hydrogel | 2022 | Rodriguez-Acosta, H. et al | Calendula extract | - | 34774393 |
|  | Gel-BA-Hydrogel | 2021 | Wang, Y. et al | Nimesulide (NIM), Vancomycin-conjugated silver nanoclusters (VAN-AgNCs) | (pro-inflammation) TNF-α↓, (anti-inflammation) IL-10↑ | 34240605 |
|  | HA-PF-Hydrogel | 2021 | Yang, H. et al | Paeoniflorin (PF) | M1(pro-inflammation) → M2 (anti-inflammation), Arg-1↑, IL-10↑, TGF-β ↑ | 34632363 |
|  | PF-127-Hydrogel | 2021 | Jiao, Y. et al | UC-MSCs | M1(pro-inflammation) → M2 (anti-inflammation) | 34717751 |
|  | GG-HA-Hydrogel | 2017 | da Silva, L. P. et al | Hyaluronic acid (HA) | M1(pro-inflammation) → M2 (anti-inflammation) | 28259681 |
|  |  |  |  |  |  |  |
| antibacterial effect | CSGI-Hydrogel | 2022 | Ji, S. et al | Macromolecular optical probe (Ir-fliq-PVP)  carboxymethyl chitosan (CMCS) | photodynamic antimicrobial chemotherapy (PACT) | 35302563 |
|  | G4-Hydrogel | 2022 | Li, Y. et al | Guanosine-quadruplex(G4) | H_2_O_2_→ROS | 35064773 |
|  | DP7-ODEX-Hydrogel | 2022 | Wu, S. et al | Peptide DP7, ceftazidime | AMP DP7 release | 34973798 |
|  | QCS-TA-Hydrogel | 2022 | Pan, W. et al | Quaternized chitosan (QCS) | QCS water-soluble release | 34896467 |
|  | QP-P-D-Hydrogel | 2022 | Wu, C. et al | Polyaniline (PANI)-grafted QCS (QCSP) | QCS and PANI release | 35288167 |
|  | Ch-AgNPs-Ce-Hydrogel | 2022 | Rodriguez-Acosta, H. et al | Silver nanoparticles (AgNPs), chitosan (CS) | AgNPs cover a greater surface area | 34774393 |
|  | nZnO-MIC-Hydrogel | 2022 | Guo, C. et al | Zinc oxide nanoparticles (nZnO) | electrostatic interactions → Zn^2+^ ↑ ROS↑ → DNA damage → destroy microbes | 35467827 |
|  | DG-Hydrogel | 2022 | Yang, J. et al. | Zinc ions | continuous infection↓ | 34879294 |
|  | PAA-CaPs-Nps@GOx-Hydrogel | 2021 | Huang, T. et al. | Fe_3_O_4_/TiO_2_/Ag_3_PO_4_ nanoparticles | H_2_O_2_→ROS | 34308944 |
|  | Gel-BA-VAN-AgNCs-Hydrogel | 2021 | Wang, Y. et al | Silver nanoclusters/ Vancomycin | Silver (Ag) →membrane destruction, ROS production, and DNA damage | 34240605 |
|  |  |  |  |  |  |  |
| antioxidant effect | MoS_2_-Au@BSA-Hydrogel | 2022 | Li, Y. et al | Bovine serum albumin (BSA) decorated Au | POD-like activity→H_2_O_2_↑，ROS↓ | 35373522 |
|  | PBNPs@PLEL-Hydrogel | 2022 | Xu, Z. et al | Prussian blue nanoparticles (PBNPs) | PBNPs → ROS↓ | 35298140 |
|  | QCS-TA-Hydrogel | 2022 | Pan, W. et al. | Tannic acid（TA） | TA→ROS↓ | 34896467 |
|  | HA-MnO_2_-FGF-2-Exos-Hydrogel | 2022 | Xiong, Y. et al | Manganese dioxide (MnO_2_) nanoenzymes | MnO_2_ induce endogenous H_2_O_2_ → O_2_ → ROS↓ | 34791802 |
|  | PEG-DA/HA-PBA/MY (PHM)-Hydrogel | 2022 | Xu, Z. et al | Myricetin (MY) | Glucose-triggered MY release → ROS↓ | 35129966 |
|  | PUAO-CPO-EXO-Hydrogel | 2020 | Shiekh, P. A. et al | Antioxidant polyurethane (PUAO) | H_2_O_2_ induced oxidative stress↓ | 32305816 |
|  |  |  |  |  |  |  |
| nerve regeneration | GM-OCS-P-Hydrogel | 2022 | Fan, L. et al | Oxidized chondroitin sulfate (OCS), OCS-polypyrrole (OCS-P) | Ca2 + ↑ → neuron proliferation and neurite outgrowth | 34648694 |
|  | rADSC-MS-Hydrogel | 2022 | Shi, M. et al | Rat adipose derived stem cells (rADSCs), rADSCs derived exosomes | β III-tubulin↑, Neurofilament-L↑ | 35433645 |
|  | ECH-Hydrogel | 2021 | Liu, C. et al | Tannic acid (TA), Polypyrrole (PPy) | MEK/ERK pathways→axonal extension, Schwann cells migration | 33937592 |
|  | HP-Hydrogel | 2018 | Li, R. et al | Nerve growth factor (NGF), Basic fibroblast growth factor (bFGF) | PI3K/Akt, JAK/STAT3 and MAPK/ERK →Schwann cells migration | 29609091 |
|  | Chitosan-Silk-Hydrogel | 2017 | Shi, Q. et al | Gingival mesenchymal stem cells (GMSCs), GMSC-derived exosomes | MSCs → nerve growth factor (NGF)↑→ neurofilaments | 29163228 |
|  | GG-HA-Hydrogel | 2017 | da Silva, L. P. et al | Human adipose-derived stem cell (hsASCs) | hASCs → Schwann cell-like phenotype →neurotrophic factors (GDNF↑, BDNF↑, CNTF↑, GFAP↑, GGF-2↑) | 28259681 |
|  |  |  |  |  |  |  |
| antiglycolytic effect | DG-Hydrogel | 2022 | Yang, J. et al | Glucose oxidase (GOx) | Glucose → H₂O₂↑ → glucose concentration↓ | 34879294 |
|  | PAA-CaPs-Nps@GOx-Hydrogel | 2021 | Huang, T. et al | Glucose oxidase (GOx) | GOx → glucose concentration↓→ ROS↑ | 34308944 |
|  | IKYLSVN-Hydrogel | 2020 | Zhao, Y. et al | Glucose oxidase (GOx) | Glucose → H₂O₂↑ → glucose concentration↓ | 31492216 |
