# Supplementary material for: Current and future outlook of loaded components in hydrogel composites for the treatment of chronic diabetic ulcers
Source: Front Bioeng Biotechnol. 2023 Feb 13;11:1077490. doi: 10.3389/fbioe.2023.1077490 (PMC9968980; doi:10.3389/fbioe.2023.1077490)
Supplement: Supplementary file 2 [file Table2.docx]

**Supplementary Table 2. Role and classification of load components in composite hydrogels**

|  | Hydrogel Composites Names  /Years | PMID  /Author | Polymer/Polysaccharide/Organic chemical | Stem cells /Exosomes/Progenitor cells | Chelating agent/Metal ion | Plant extracts | Proteins (Cytokines /Peptides/Enzymes) and Nucleoside products | Medicine/Drugs |
| --- | --- | --- | --- | --- | --- | --- | --- | --- |
| vascular regeneration/angiogenesis | OSA-DA-Hydrogel  2022 | 35101479  Chi, J. et al. | Dopamine (DA) |  |  |  |  |  |
|  | Q-P-D-Hydrogel  2022 | 35288167  Wu, C. et al. |  |  | Deferoxamine (DFO) |  |  |  |
|  | DG-Hydrogel  2022 | 34879294  Yang, J. et al. |  |  | Deferoxamine (DFO) |  |  |  |
|  | SA-DFO-Cu-Hydrogel  2022 | 35066024  Li, S. et al |  |  | Deferoxamine (DFO), copper nanoparticles (Cu-NPs) |  |  |  |
|  | SC-Ps-sEVs-Hydrogel  2022 | 35156747  Ma, S. et al. |  | Umbilical cord mesenchymal stem cells (ucMSCs)-derived exosomes |  |  |  |  |
|  | HA-MnO_2_-FGF-2-Exos-Hydrogel  2022 | 34791802  Xiong, Y. et al |  | M2-derived Exosomes (M2 Exos) |  |  | FGF-2 growth factor |  |
|  | GelMA-PEGDA-MN-exos-Hydrogel  2022 | 35305648  Yuan, M. et al |  | HUVECs-exos |  |  |  | Tazarotene |
|  | siNP-BG-SA-Hydrogel  2022 | 35421617  Li, Y. et al |  |  |  |  | Small interfering RNA of MMP9 (MMP9-siRNA) |  |
|  | GM-OCS-P-Hydrogel  2022 | 34648694  Fan, L. et al | Oxidized chondroitin sulfate -polypyrrole (OCS-P) |  |  |  |  |  |
|  | EPCs-aFGF@GelMA-Hydrogel  2022 | 34973992  Zhu, H. et al |  | Endothelial progenitor cells (EPCs) |  |  | Acid fibroblast growth factor (aFGF) |  |
|  | nZnO-MIC-Hydrogel  2022 | 35467827  Guo, C. et al |  |  |  | Paeoniflorin-encapsulated micelle (MIC) |  |  |
|  | P_E_-E_NP_-PCH-Hydrogel  2022 | 35335913  Lee, Y.H. et al |  |  |  |  | Epidermal Growth Factor (EGF) |  |
|  | P-Exos-CMC  2021 | 34706802  Huang, L. et al |  | Plasma exosomes(P-Exos) |  |  |  |  |
|  |  |  |  |  |  |  |  |  |
| anti-inflammatory effect | P_E_-E_NP_-PCH-Hydrogel  2022 | 35335913  Lee, Y.H. et al | Chitosan |  |  |  |  |  |
|  | siNP-BG-SA-Hydrogel  2022 | 35421617  Li, Y. et al | Bioglass (BG) |  |  |  |  |  |
|  | B-G-Hydrogel  2022 | 35319815  Liu, J. et al |  |  |  | Bletilla Striata polysaccharide (BSP) |  |  |
|  | Ch-AgNPs-Ce-Hydrogel  2022 | 34774393  Rodriguez-Acosta, H. et al |  |  |  | Calendula extract |  |  |
|  | Gel-BA-VAN-AgNCs-Hydrogel  2021 | 34240605  Wang, Y. et al |  |  |  |  |  | Nimesulide (NIM) |
|  | HA-PF-Hydrogel  2021 | 34632363  Yang, H. et al |  |  |  | Paeoniflorin (PF) |  |  |
|  | PF-127-Hydrogel  2021 | 34717751  Jiao, Y. et al |  | Umbilical cord mesenchymal stem cells (ucMSCs)-derived exosomes |  |  |  |  |
|  | GG-HA-Hydrogel  2017 | 28259681  da Silva, L. P. et al |  | Human adipose stem cells (hASCs) |  |  |  |  |
|  |  |  |  |  |  |  |  |  |
| antibacterial effect | CSGI-Hydrogel  2022 | 35302563  Ji, S. et al | Carboxymethyl chitosan (CMCS) |  | Macromolecular optical probe (Ir-fliq-PVP) |  |  |  |
|  | G4-Hydrogel  2022 | 35064773  Li, Y. et al |  |  |  |  | Guanosine-quadruplex(G4) |  |
|  | DP7-ODEX-Hydrogel  2022 | 34973798  Wu, S. et al |  |  |  |  | Peptide DP7 | Ceftazidime |
|  | QCS-TA-Hydrogel  2022 | 34896467  Pan, W. et al | Quaternized chitosan (QCS) |  |  | Tannic acid (TA) |  |  |
|  | Q-P-D-Hydrogel  2022 | 35288167  Wu, C. et al | Polyaniline-grafted quaternized chitosan (QCS-P) |  |  |  |  |  |
|  | Ch-AgNPs-Ce-Hydrogel  2022 | 34774393  Rodriguez-Acosta, H. et al | Chitosan (CS) |  | Silver nanoparticles (AgNPs) |  |  |  |
|  | nZnO-MIC-Hydrogel  2022 | 35467827  Guo, C. et al |  |  | Zinc oxide nanoparticles (nZnO) |  |  |  |
|  | DG-Hydrogel  2022 | 34879294  Yang, J. et al. |  |  | Zinc ions |  |  |  |
|  | PAA-CaPs-Nps@GOx-Hydrogel  2021 | 34308944  Huang, T. et al. |  |  | Fe_3_O_4_/TiO_2_/Ag_3_PO_4_ nanoparticles |  |  |  |
|  | Gel-BA-VAN-AgNCs-Hydrogel  2021 | 34240605  Wang, Y. et al |  |  | Silver (Ag) nanoclusters |  |  | Vancomycin |
|  |  |  |  |  |  |  |  |  |
| antioxidant effect | MoS_2_-Au@BSA-Hydrogel  2022 | 35373522  Li, Y. et al |  |  | Bovine serum albumin (BSA) decorated Au |  |  |  |
|  | PBNPs@PLEL-Hydrogel  2022 | 35298140  Xu, Z. et al |  |  | Prussian blue nanoparticles (PBNPs) |  |  |  |
|  | QCS-TA-Hydrogel  2022 | 34896467  Pan, W. et al. |  |  |  | Tannic acid (TA) |  |  |
|  | HA-MnO_2_-FGF-2-Exos-Hydrogel  2022 | 34791802  Xiong, Y. et al |  |  | Manganese dioxide (MnO_2_) nanoenzymes |  |  |  |
|  | PEG-DA/HA-PBA/MY (PHM)-Hydrogel  2022 | 35129966  Xu, Z. et al |  |  |  | Myricetin (MY) |  |  |
|  | PUAO-CPO-EXO-Hydrogel  2020 | 32305816  Shiekh, P. A. et al | antioxidant polyurethane (PUAO) |  |  |  |  |  |
|  |  |  |  |  |  |  |  |  |
| nerve regeneration | GM-OCS-P-Hydrogel  2022 | 34648694  Fan, L. et al | Oxidized chondroitin sulfate-polypyrrole (OCS-P) |  |  |  |  |  |
|  | rADSC-MS-Hydrogel  2022 | 35433645  Shi, M. et al |  | Rat adipose derived stem cells (rADSCs)  rADSCs derived exosomes |  |  |  |  |
|  | ECH-Hydrogel  2021 | 33937592  Liu, C. et al | Polypyrrole (PPy) |  |  |  |  |  |
|  | HP-Hydrogel  2018 | 29609091  Li, R. et al |  |  |  |  | Nerve growth factor (NGF)  basic fibroblast growth factor (bFGF) |  |
|  | Chitosan-Silk-Hydrogel  2017 | 29163228  Shi, Q. et al |  | Human adipose-derived stem cell (hsASCs) |  |  |  |  |
|  | GG-HA-Hydrogel  2017 | 28259681  da Silva, L. P. et al |  | Human adipose-derived stem cell (hsASCs) |  |  |  |  |
|  |  |  |  |  |  |  |  |  |
| antiglycolytic effect | DG-Hydrogel  2022 | 34879294  Yang, J. et al |  |  |  |  | Glucose oxidase (GOx) |  |
|  | PAA-CaPs-Nps@GOx-Hydrogel  2021 | 34308944  Huang, T. et al |  |  |  |  | Glucose oxidase (GOx) |  |
|  | IKYLSVN-Hydrogel  2020 | 31492216  Zhao, Y. et al |  |  |  |  | Glucose oxidase (GOx) |  |
